# Targeting radioresistance and replication fork stability in prostate cancer

**DOI:** 10.1172/jci.insight.152955

**Published:** 2022-05-09

**Authors:** Xiangyi Li, GuemHee Baek, Suzanne Carreira, Wei Yuan, Shihong Ma, Mia Hofstad, Sora Lee, Yunpeng Gao, Claudia Bertan, Maria de los Dolores Fenor de la Maza, Prasanna G. Alluri, Sandeep Burma, Benjamin P.C. Chen, Ganesh V. Raj, Johann de Bono, Yves Pommier, Ram S. Mani

**Affiliations:** 1 Department of Pathology, University of Texas (UT) Southwestern Medical Center, Dallas, Texas, USA.; 2 Prostate Cancer Targeted Therapy and Cancer Biomarkers Group, The Institute of Cancer Research and The Royal Marsden National Health Service (NHS) Foundation Trust, Sutton, United Kingdom.; 3 Department of Urology and; 4 Department of Radiation Oncology, UT Southwestern Medical Center, Dallas, Texas, USA.; 5 Department of Biochemistry and Structural Biology and Department of Neurosurgery, UT Health Science Center, San Antonio, Texas, USA.; 6 Developmental Therapeutics Branch, Center for Cancer Research, National Cancer Institute, NIH, Bethesda, Maryland, USA.; 7 Harold C. Simmons Comprehensive Cancer Center, UT Southwestern Medical Center, Dallas, Texas, USA.

**Keywords:** Oncology, Cancer, Radiation therapy, Urology

## Abstract

The bromodomain and extraterminal (BET) family of chromatin reader proteins bind to acetylated histones and regulate gene expression. The development of BET inhibitors (BETi) has expanded our knowledge of BET protein function beyond transcriptional regulation and has ushered several prostate cancer (PCa) clinical trials. However, BETi as a single agent is not associated with antitumor activity in patients with castration-resistant prostate cancer (CRPC). We hypothesized novel combinatorial strategies are likely to enhance the efficacy of BETi. By using PCa patient-derived explants and xenograft models, we show that BETi treatment enhanced the efficacy of radiation therapy (RT) and overcame radioresistance. Mechanistically, BETi potentiated the activity of RT by blocking DNA repair. We also report a synergistic relationship between BETi and topoisomerase I (TOP1) inhibitors (TOP1i). We show that the BETi OTX015 synergized with the new class of synthetic noncamptothecin TOP1i, LMP400 (indotecan), to block tumor growth in aggressive CRPC xenograft models. Mechanistically, BETi potentiated the antitumor activity of TOP1i by disrupting replication fork stability. Longitudinal analysis of patient tumors indicated that *TOP1* transcript abundance increased as patients progressed from hormone-sensitive prostate cancer to CRPC. *TOP1* was highly expressed in metastatic CRPC, and its expression correlated with the expression of BET family genes. These studies open new avenues for the rational combinatorial treatment of aggressive PCa.

## Graphical abstract

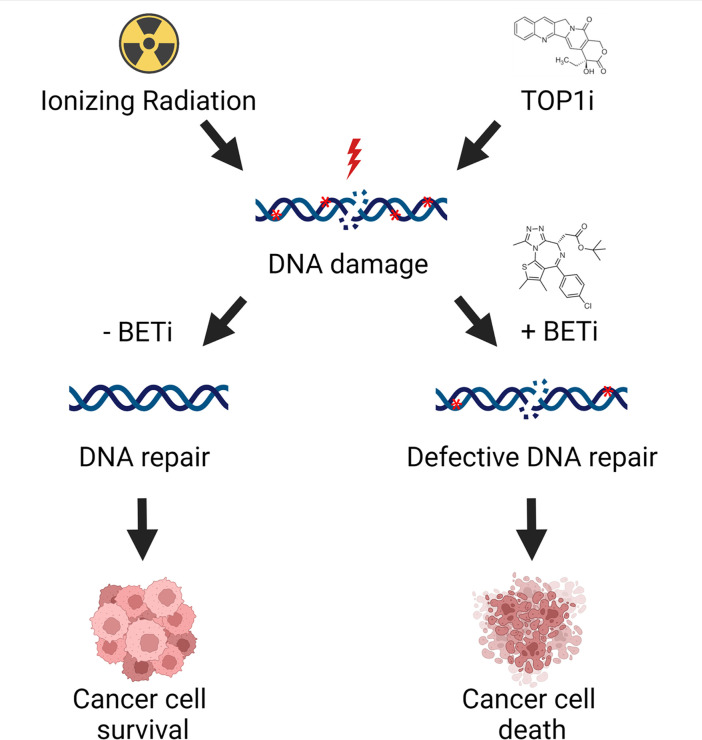

## Introduction

Prostate cancer (PCa) is the most commonly diagnosed cancer in men, accounting for 26% of cancer diagnoses (1). Despite this high incidence, the 5-year survival rate for localized PCa is 98%. This is in part due to advances in treatment modalities. Other reasons include the indolent nature of these diseases in many patients and improved stratification of patients with PCa into clinical risk groups (low, intermediate, and high risk), which has allowed deintensification of treatment in favorable-risk patients and escalation of treatment in high-risk patients. PCa is associated with relatively low rates of point mutations and higher rates of chromosomal aberrations, including structural variants and genomic rearrangements (2–4). Clonal genomic rearrangements and fusions resulting in upregulation of ETS transcription factor family genes — *ERG* and *ETV1* — occur in approximately 50% of PCa cases (5). Aberrant signaling of the androgen receptor (AR) drives many facets of PCa etiology, including initiation of lethal metastatic castration-resistant prostate cancer (mCRPC) (6). Copy number alterations and amplifications of the AR locus are observed in more than 60% of mCRPCs (7, 8).

Inflammation is a risk factor for prostate carcinogenesis (9). PCa development is associated with crosstalk between epithelial cells and the surrounding stroma. The tumor microenvironment influences metastasis via integrins and extracellular proteases, among others (10). A significant proportion of men with localized PCa progress to metastatic PCa, and many present with advanced disease, which is invariably lethal (11). Enhancing the efficacy of treatments for localized PCa — including radiation therapy (RT) — can delay or prevent the emergence of metastatic disease. Men with metastatic PCa initially respond to drugs that block androgen biosynthesis (e.g., abiraterone) or inhibit AR activity (e.g., enzalutamide) but eventually progress to mCRPC. Resistance to androgen signaling inhibitors involves multiple mechanisms, including AR locus amplification, the formation of AR variants, induction of glucocorticoid receptor expression, and lineage switch, among others (12). A subset of mCRPC patients with disease progression while receiving enzalutamide or abiraterone respond to the PARP inhibitor (PARPi) olaparib (13–15). Tumor cell defects in homologous recombination (HR) repair genes predict response to PARPi. Given that HR repair defects are only observed in a small percentage of men with mCRPC, there is a critical need to develop new single-agent or combination therapies for HR repair–proficient tumors.

The bromodomain and extraterminal (BET) family proteins — BRD4, BRD3, BRD2, and BRDT — bind to acetylated histones and regulate gene expression (16). BRD4, BRD3, and BRD2 are ubiquitously expressed, whereas the expression of BRDT is restricted to the male germ cells. BRD4 is the best studied member of the BET family of chromatin reader proteins. The development of BET inhibitors (BETi) has enhanced our understanding of the role of BET proteins in genome regulation and led to more than 20 clinical trials (17–20). The BETi JQ1 binds competitively to the acetyl-lysine recognition motifs, termed bromodomains, in the BET proteins (21). The next-generation BETi, dBET1, synthesized by the conjugation of JQ1 with pthalimide moiety, induces selective degradation of BET proteins (22). The JQ1 analog, OTX015, is a competitive oral inhibitor of BET proteins and is suitable for human use (23–25).

In addition to transcription control, BET proteins have been implicated in the regulation of DNA repair. Prior studies by our group and others have shown that BET proteins are essential for the repair of DNA double-strand breaks (DSBs) by nonhomologous end joining (NHEJ) (26, 27). BET proteins also promote the repair of DNA DSBs by HR (28–30). Furthermore, BET proteins have also been implicated in regulating proliferating cell nuclear antigen unloading during DNA replication, in R-loop suppression, and in resolving conflicts between transcription and DNA replication (31–35). However, BETi as a single-agent treatment in phase I clinical trials has not been associated with antitumor activity in CRPC patients due to limited tolerability from repeated dosing (36–38). Interestingly, the combination of BETi and enzalutamide demonstrates acceptable tolerability and some antitumor activity in patients with androgen signaling inhibitor–resistant mCRPC (39, 40). Taken together, these studies support the pursuit of rational combinatorial strategies with BETi for treating lethal PCa.

We present “mechanism-guided” combinatorial strategies with BETi to enhance the efficacy of PCa RT and targeted therapy. We have shown that the expression of BRD4 in pretreatment PCa biopsies is negatively associated with outcome after RT, indicating that BETi can be employed as radiosensitizers (26). In this study, we leverage patient-derived explants (PDEs) and xenograft models to demonstrate the role of BETi in enhancing the efficacy of RT and overcoming radioresistance. Inspired by the synthetic lethal interaction between BETi and chemotherapeutic agents, such as PARPi, we sought to identify additional such interactions (28–30, 41). We demonstrate a synthetic lethal interaction between BETi and topoisomerase I (TOP1) inhibitors (TOP1i) by employing in vivo tumor models. Mechanistically, BETi synergizes with TOP1i to impair the stability of DNA replication forks during cell division. These findings open new avenues for BETi combination therapy in the treatment of mCRPC.

## Results

### BETi enhances the efficacy of radiotherapy in PDE and xenograft models.

**Figure 1:**
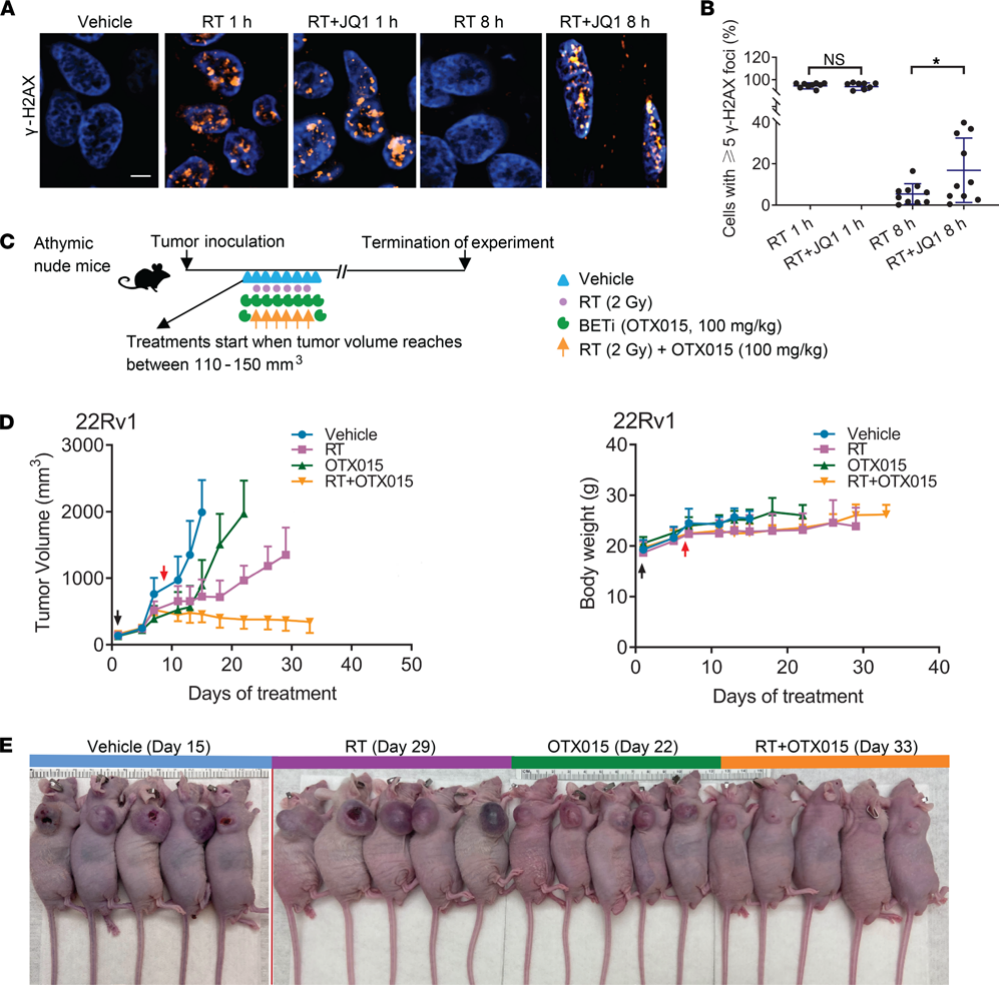
BETi enhances the efficacy of RT. (**A**) γ-H2AX foci (original magnification, 60×) immunofluorescence staining with PDEs. Scale bar: 10 μm. (**B**) γ-H2AX foci quantification in the 4 treatment groups (*P < 0.05; 2-tailed Student’s *t* test). (**C**) Subcutaneous tumor xenograft treatment schematic. OTX015 dose: 100 mg/kg, oral gavage, 1 dosage per day; RT dose: 2 Gy per day. (**D**) Left panel, growth of 22Rv1 tumor xenografts with indicated treatments. Black and red arrows indicate treatment start and treatment endpoints, respectively. The *P* values were obtained from unpaired 2-tailed Student’s *t* test. The *P* values were adjusted by Benjamini-Hochberg procedure for multiple comparisons. *n* ≥ 5 mice per group. Means ± SEM. Note that half of the error bars are shown here to facilitate clear view. The *P* values are listed in Supplemental Table 2. Right panel, measurement of mice body weight. (**E**) Representative images of mice from all treatment arms. The vertical red lines separate individual images. Color bars on top represent the treatment groups. The number of days inside of parentheses indicates the total time from the start of treatment to experiment termination. The images were obtained on the day of experiment termination.

We explored the role of BETi in enhancing the efficacy of irradiation in PDEs obtained from radical prostatectomy specimens from patients with clinically localized PCa (Supplemental Table 1; supplemental material available online with this article; https://doi.org/10.1172/jci.insight.152955DS1). The PDEs displayed morphological features consistent with cancer (H&E) and stained positive for pan-Cytokeratin (Supplemental Figure 1, A–C). In these model systems (42), DNA damage induced by 2 Gy ionizing radiation (IR) was largely repaired within 8 hours as indicated by the reduction in γ-H2AX signal, representing phosphorylated histone H2AX (Ser139) (Figure 1A). Treatment with the BETi JQ1 blocked the repair of IR-induced DNA DSBs as indicated by the persistence of γ-H2AX signal at 8 hours (Figure 1B). These results indicate the potential utility of BETi as a radiosensitizer.

Androgen deprivation therapy (ADT) is increasingly used in combination with RT for the treatment of high-risk localized PCa. However, despite initial response, many tumors eventually develop resistance to ADT-RT combination. This is, in part, mediated by the formation of AR variants (AR-Vs), which promote DNA repair in an ADT-independent manner (43). Thus, there is a critical need to enhance the efficacy of RT in ADT-resistant tumor models expressing AR-Vs. The 22Rv1 PCa cells express AR-Vs, are resistant to androgen signaling inhibitors, and are only partially responsive to IR. Prior work by our group demonstrated that AR-Vs mediate DNA repair after RT (43). We have also shown that BETi treatment downregulated the expression of AR and AR-Vs, in addition to blocking DNA repair (18, 26, 44). Therefore, we sought to test the role of BETi as radiosensitizer.

We used the BETi OTX015 for our in vivo studies because this inhibitor can be administered orally and is currently being evaluated in multiple clinical trials. Consistent with JQ1 treatment (26), treatment with the BETi OTX015 blocked NHEJ DNA repair (Supplemental Figure 1D). We explored the utility of OTX015 in enhancing the efficacy of RT in the 22Rv1 xenograft model system. To mimic the clinical practice of patients receiving fractionated radiation over several days, we treated mice with radiation utilizing a 6-day regimen (2 Gy/d) (Figure 1C). Single-agent OTX015 was administered for 8 days. For the combination treatment, OTX015 was started 1 day prior to initiating RT, administered along with RT for 6 days, and administered for 1 day after completion of the RT treatment course. Single-agent RT treatment reduced tumor growth whereas single-agent OTX015 treatment did not influence tumor growth (Figure 1, D and E, and Supplemental Table 2). The combination of RT and OTX015 had superior antitumor activity to RT alone against 22Rv1 tumors. The single-agent and combination treatments were not associated with noticeable changes in body weight, thereby indicating good tolerability. These results signify the potential of BETi in enhancing the efficacy of RT in the treatment of aggressive cancers.

### Treatment with BETi reverses radioresistance.

**Figure 2:**
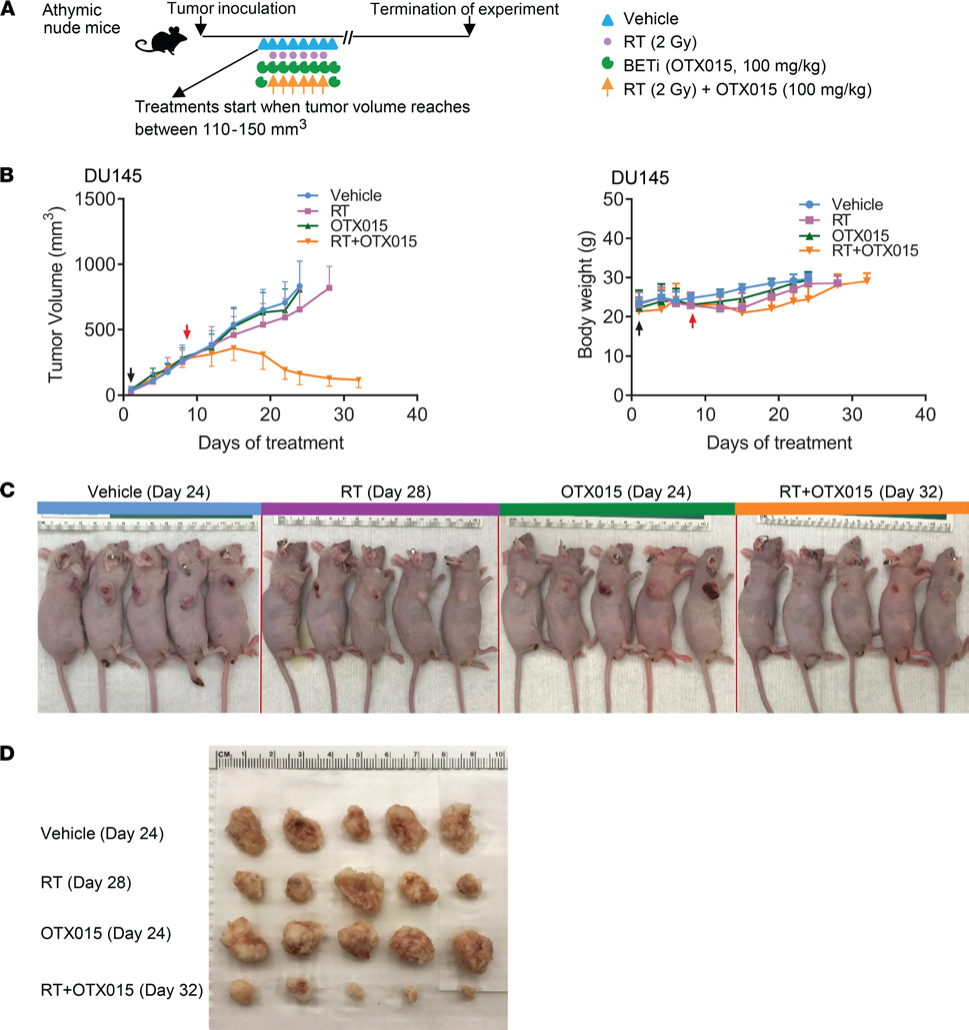
Targeting radioresistance with BETi. (**A**) Subcutaneous tumor xenograft treatment schematic. OTX015 dose: 100 mg/kg, oral gavage, 1 dosage per day; RT dose: 2 Gy per day. (**B**) Left panel, growth of DU145 tumor xenografts with indicated treatments. Black and red arrows indicate treatment start and treatment endpoints, respectively. The *P* values were obtained from unpaired 2-tailed Student’s *t* test. The *P* values were adjusted by Benjamini-Hochberg procedure for multiple comparisons. *n* ≥ 5 mice per group. Means ± SEM. Note that half of the error bars are shown here to facilitate clear view. The *P* values are listed in Supplemental Table 3. Right panel, measurement of mice body weight. (**C**) Representative images of mice from all treatment arms. The vertical red lines separate individual images. Color bars on top represent the treatment groups. The number of days inside of parentheses indicate the total time from the start of treatment to experiment termination. The images were obtained on the day of experiment termination. (**D**) Image of dissected xenografts from all treatment groups.

We next explored the utility of BETi in overcoming radioresistance (Figure 2A). The DU145 tumor model, representing aggressive CRPC, does not express AR and is radiation resistant. Prior studies indicated that treatment of DU145 cells with BETi as a single-agent had minimal effect on their proliferation (17). We observed that DU145 xenograft tumors did not respond to single-agent RT treatment or single-agent OTX015 treatment. Remarkably, however, the combination of RT and OTX015 had impressive antitumor activity (Figure 2, B–D, and Supplemental Table 3). The single-agent and combination treatments were not associated with noticeable changes in mouse body weight, indicating good tolerability in the animals. It is well established that treatment with BETi interferes with both transcriptional regulation and the repair of DNA DSBs. Given that the single-agent treatment with BETi had no effect on tumor growth, we hypothesize that the observed effects of BETi in this combination treatment (BETi + RT) are largely due to impairment of DNA repair. These results suggest that utilizing an intermittent BETi schedule can have benefit in treating radioresistant PCa.

### BETi synergizes with TOP1i to disrupt replication fork stability.

Given the emerging role of BET proteins in HR DNA repair (28–30), we sought to develop novel treatment strategies. We therefore verified the impact of BETi treatment on HR DNA repair by flow cytometry analysis of engineered HEK293T cells harboring the I-SceI-DR-GFP reporter. Treatment with JQ1 or OTX015 resulted in a reduction in HR activity (Supplemental Figure 2, A–D). Consistent with these observations, siRNA-based knockdown of BRD4, BRD3, or BRD2 also resulted in a reduction in HR activity (Supplemental Figure 2, E and F). We assessed the generalizability of these results by conducting quantitative PCR–based (QPCR-based) HR assay in 4 PCa cell lines (LNCaP, VCaP, 22Rv1, and DU145). Treatment with JQ1 reduced HR activity in these 4 cell lines (Supplemental Figure 3A). These results were also recapitulated by siRNA-based knockdown of BRD4, BRD3, and BRD2, individually and in combination (Supplemental Figure 3, B and C).

Treatment of U2OS cells with JQ1 impaired IR-induced RAD51 foci formation in a dose-dependent manner, thereby providing a mechanistic rationale for compromised HR DNA repair (Supplemental Figure 4, A–C). We extended our studies to dBET1, which induces selective degradation of BET proteins (22). Analogous to JQ1 treatment, treatment with dBET1 impaired IR-induced RAD51 foci formation in a dose-dependent manner (Supplemental Figure 4, D and E). These results were recapitulated by siRNA-based knockdown of BRD4, BRD3, and BRD2, individually and in combination (Supplemental Figure 4F). Furthermore, overexpression of BRD4 resulted in an increase in IR-induced RAD51 foci formation (Supplemental Figure 4G). Mechanistically, treatment with JQ1 resulted in the downregulation of RAD51 in a dose-dependent manner (Supplemental Figure 4H). Analysis of published ChIP-Seq data sets (17) indicated that JQ1 treatment blocked the recruitment of BRD4, BRD3, and BRD2 to the +1 nucleosome relative to the *RAD51* transcription start site (TSS) in VCaP cells and concomitantly reduced the occupancy of RNA polymerase II at the TSS (Supplemental Figure 4I), suggesting transcriptional downregulation.

Based on these observations, we hypothesized that BETi can potentially be used to target replication fork stability. TOP 1 is essential for releasing supercoils arising in front of the moving replication fork during DNA replication (45). The TOP1i camptothecin (CPT) traps TOP1 with DNA, resulting in the formation of trapped TOP1 cleavage complexes, which impede DNA uncoiling (46). This can lead to multiple consequences for the replicating cell. For example, the TOP1 cleavage complex can contribute to DNA replication fork stalling and reversal, protective mechanisms that limit the formation of DNA DSBs (47, 48). RAD51 promotes fork reversal and is considered a fork protection enzyme (49, 50). Collision of the moving DNA replication fork with the trapped TOP1 cleavage complex can result in the formation of single-ended DSBs, which are predominantly repaired by the HR DNA repair pathway (51). Given the established role of BETi in blocking DNA repair, and regulating RAD51 expression, we conducted DNA fiber assays to test the role of BET proteins in replication fork stability upon CPT treatment.

**Figure 3:**
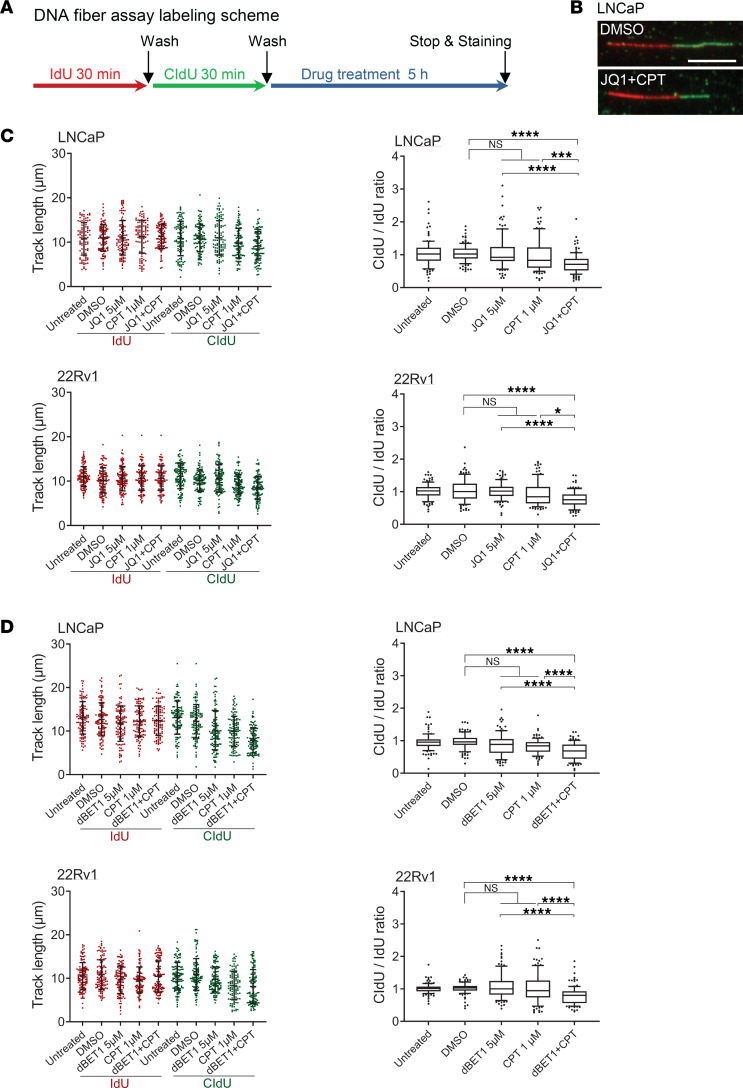
BETi synergizes with TOP1i to disrupt replication fork stability. (**A**) Labeling scheme. Cells were pulse labeled first with IdU (100 μM, 30 minutes) and then with CldU (100 μM, 30 minutes), followed by drug treatment for 5 hours. (**B**) Representative images of DNA tracks (fork direction: red to green). Scale bar: 10 μm. (**C** and **D**) The effect of treatment with BETi (JQ1 or dBET1) and/or TOP1i (CPT) on DNA replication fork stability. The length of labeled DNA tracks, IdU (red) and CldU (green), was scored (*n* ≥ 110) and displayed in scatter dot plots (left panels). CldU/IdU ratio of each track was calculated and displayed in box-and-whisker plots (right panels). Boxes: 25th–75th percentile; whiskers: 10th–90th percentile. Two-tailed Mann-Whitney *U* test was applied; the *P* values were adjusted by Benjamini-Hochberg procedure for multiple comparisons (****P < 0.0001; ***P < 0.001; *P < 0.05). One of the 2 independent replicates is shown.

DNA fiber assays were performed in LNCaP cells, which are androgen responsive, as well as the 22Rv1 cells representing CRPC. The cells were sequentially pulse labeled with iodo-deoxyuridine (IdU; shown in red) and chloro-deoxyuridine (CldU; shown in green) to track the direction of replication fork movement (forks proceed from red to green), followed by treatment with various drug combinations (Figure 3A). A CldU/IdU ratio of 1 indicates no treatment effect on replication fork stability. We observed that single-agent treatment with CPT, JQ1, or dBET1 had a modest effect on replication fork stability in LNCaP and 22Rv1 cells. Strikingly, the combination treatment of CPT and BETi (JQ1 or dBET1) resulted in a significant reduction in replication fork stability (Figure 3, B–D). The CldU/IdU ratio for the combination treatment was significantly lower than the single-agent treatments or vehicle (DMSO) treatment in both the LNCaP and 22Rv1 cells. These results demonstrate that TOP1i and BETi can synergize to destabilize replication forks.

**Figure 4:**
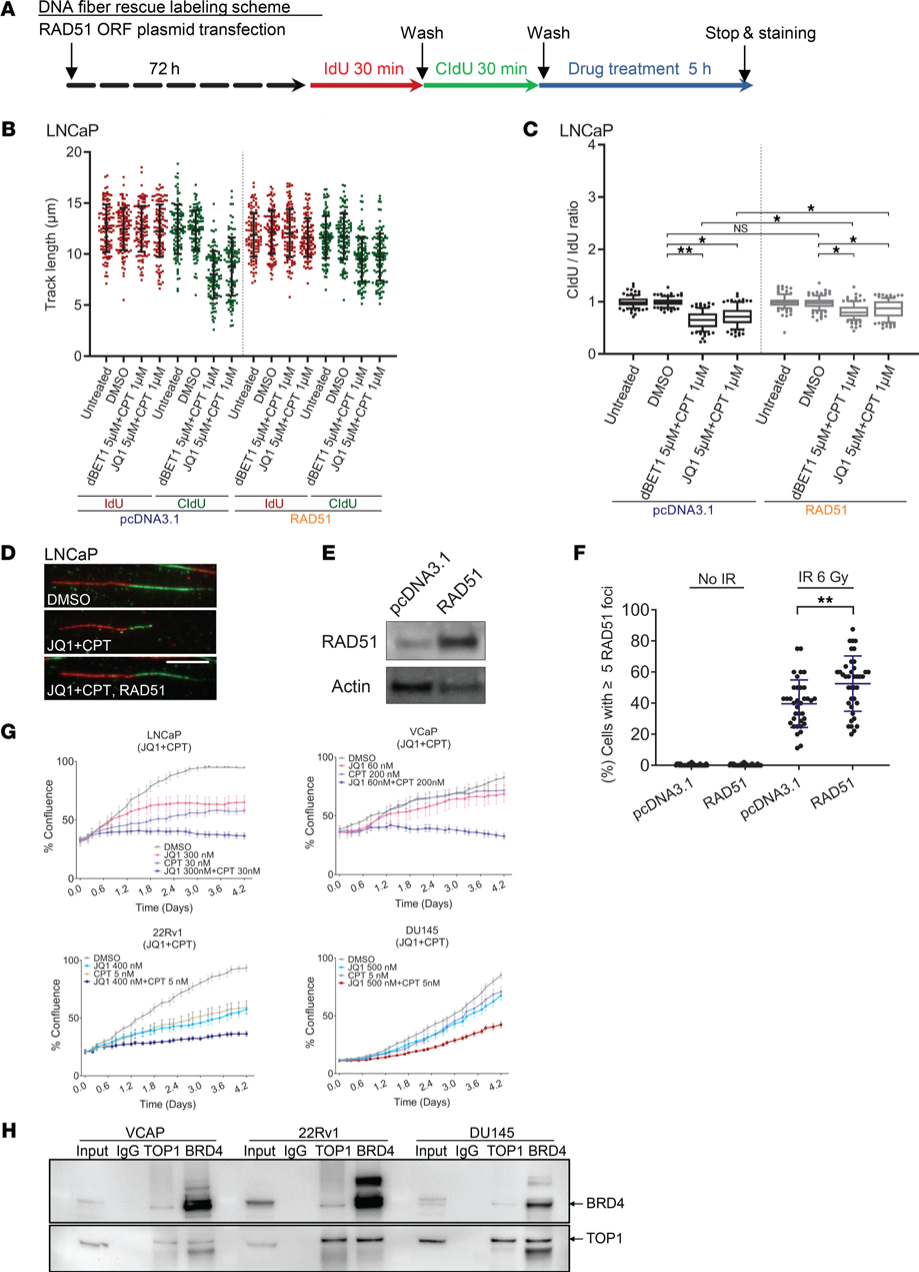
Overexpression of RAD51 partially rescues the effect of BETi-TOP1i combination treatment on replication fork stability. (**A**) Labeling scheme. Cells were transfected with control or RAD51 overexpression plasmid (72 hours), then were pulse labeled first with IdU (100 μM, 30 minutes) and then with CldU (100 μM, 30 minutes), followed by drug treatment for 5 hours. (**B** and **C**) The length of labeled DNA tracks, IdU (red) and CldU (green), was scored (*n* ≥ 110) and displayed in scatter dot plots (left panel). CldU/IdU ratio of each track was calculated and displayed in box-and-whisker plots (right panel). Boxes: 25th–75th percentile; whiskers: 10th–90th percentile. Two-tailed Mann-Whitney *U* test was applied; the *P* values were adjusted by Benjamini-Hochberg procedure for multiple comparisons (**P < 0.01; *P < 0.05). (**D**) Representative images of DNA tracks (fork direction: red to green). Scale bar: 10 μm. (**E**) Immunoblot verification of RAD51 overexpression upon plasmid transfection in LNCaP cells (72 hours). (**F**) RAD51 foci were analyzed in U2OS cells upon RAD51 overexpression with or without irradiation (IR 6 Gy). pcDNA3.1 was used as a mock control. Cells were analyzed 4 hours after IR treatment. Cells with ≥5 foci were counted. **P < 0.01, Mann-Whitney *U* test. (**G**) The effect of treatment with JQ1 and/or CPT on the proliferation of LNCaP, VCaP, 22Rv1, and DU145 PCa cells. Two-tailed Student’s *t* test was applied; the *P* values were adjusted by Benjamini-Hochberg procedure for multiple comparisons; error bars, SEM of 3 technical triplicates. The *P* values are listed in Supplemental Table 4. (**H**) Immunoprecipitation (IP) was performed using BRD4 or TOP1 antibodies in VCaP, 22Rv1, and DU145 cells and analyzed by immunoblot with the indicated antibodies.

To determine whether the observed effects of BETi on the stability of replication forks upon TOP1i treatment were due to RAD51 downregulation, we conducted rescue experiments in LNCaP cells (Figure 4A). Overexpression of RAD51 partially rescued the reduction in replication fork stability phenotype, thereby providing a mechanistic rationale for the TOP1i-BETi combination. The CldU/IdU ratio for the combination treatment with RAD51 overexpression was significantly higher than the combination treatment without RAD51 overexpression, indicating an enhancement in replication fork stability (Figure 4, B–E). RAD51 overexpression in U2OS cells resulted in increased RAD51 transcript abundance (Supplemental Figure 5A) and increased IR-induced RAD51 foci formation (Figure 4F and Supplemental Figure 5B). Furthermore, RAD51 overexpression increased HR activity in 4 PCa cell lines (LNCaP, VCaP, 22Rv1, and DU145) (Supplemental Figure 5, C and D). BETi treatment resulted in the transcriptional downregulation of additional DNA repair genes, including BRCA1, BRCA2, WRN, NBN, and MRE11, in multiple cell lines (Supplemental Figure 6). Analysis of published ChIP-Seq data sets (17) indicated that JQ1 treatment reduced the occupancy of RNA polymerase II at the TSS of these genes in VCaP cells (Supplemental Figure 6), confirming transcriptional downregulation. We also observed that combination of CPT and JQ1 was potent in blocking the proliferation of LNCaP, VCaP, 22Rv1, and DU145 PCa cells (Figure 4G, Supplemental Figure 7, and Supplemental Table 4). Coimmunoprecipitation experiments with BRD4 antibodies indicated interaction with TOP1 in VCaP, 22Rv1, and DU145 (Figure 4H). Similarly, coimmunoprecipitation experiments with TOP1 antibodies indicated interaction with BRD4 in the same 3 cell lines (Figure 4H). Taken together, these results point to the utility of combining BETi and TOP1i in the systemic treatment of metastatic PCa.

### OTX015 potentiates the antitumor activity of LMP400.

**Figure 5:**
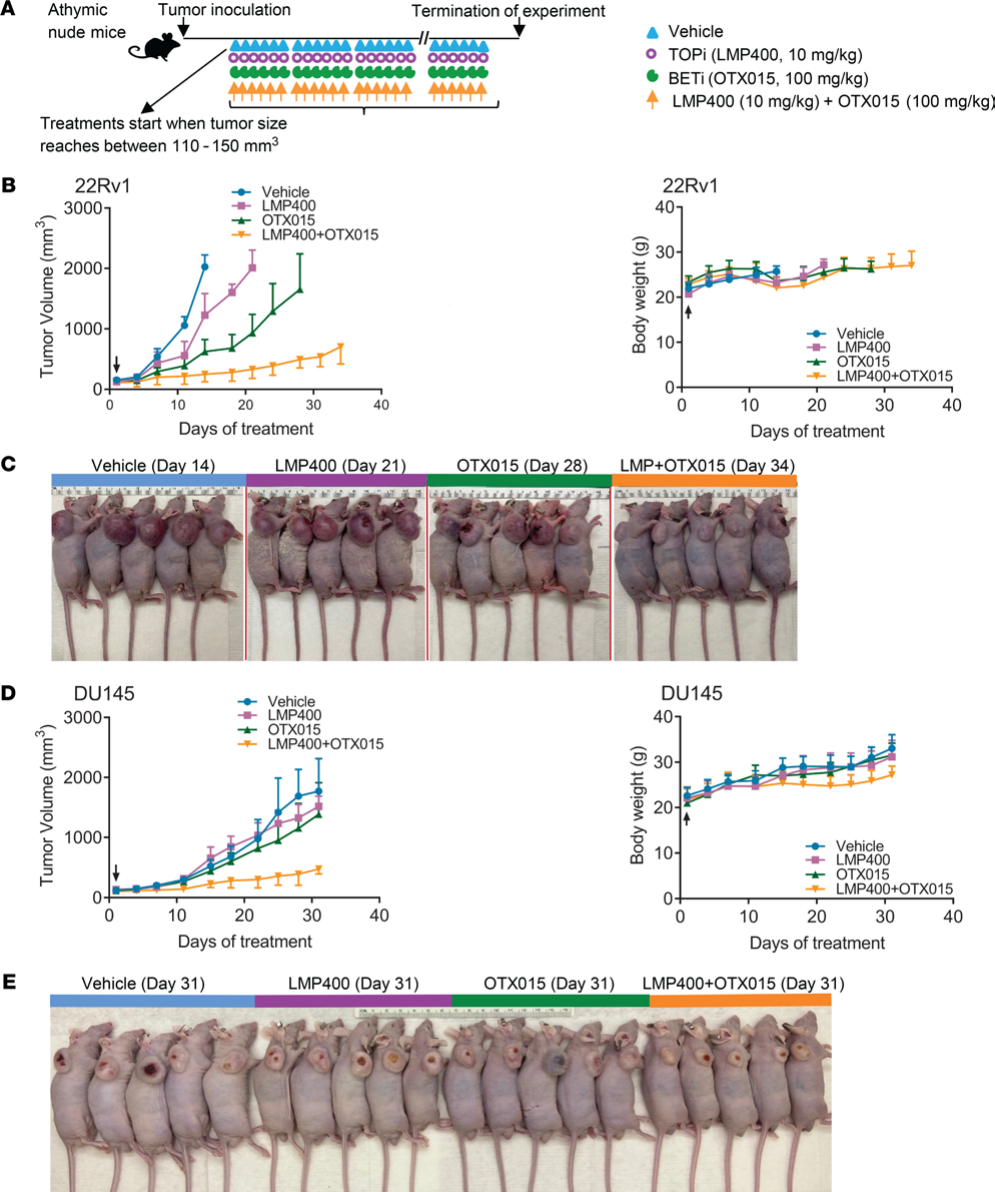
OTX015 potentiates the antitumor activity of LMP400 in subcutaneous 22Rv1 and DU145 tumor xenografts. (**A**) Subcutaneous tumor xenograft treatment schematic. OTX015 dose: 100 mg/kg, oral gavage, 1 dosage per day; LMP400 dose: 10 mg/kg, IP, once per day. Xenograft tumor-bearing mice were continuously treated (6-day-on/1-day-off cycle) with vehicle or LMP400 or OTX015 or LMP400 + OTX015 until experiment termination. (**B**) Left panel, growth of 22Rv1 tumor xenografts with indicated treatments. Black arrow indicates treatment start point. The *P* values were obtained from unpaired 2-tailed Student’s *t* test. The *P* values were adjusted by Benjamini-Hochberg procedure for multiple comparisons. *n* ≥ 5 mice per group. Means ± SEM. Note that half of the error bars are shown here to facilitate clear view. Right panel, measurement of mice body weight. (**C**) Representative images of mice from all treatment arms. The vertical red lines separate individual images. Color bars on top represent the treatment groups. The number of days inside of parentheses indicates the total time from the start of treatment to experiment termination. The images were obtained on the day of experiment termination. (**D**) Same as **B** but with DU145 tumor xenografts. (**E**) Same as **C** but with DU145 tumor xenografts. The *P* values are listed in Supplemental Tables 5 and 6.

CPT and its derivatives, such as irinotecan, topotecan, and belotecan, have limited utility in the clinical setting because of their inherent chemical instability, short half-life, reversibility of TOP1 cleavage complexes, and drug efflux by ABCG2, which decreases intracellular drug concentrations (52). LMP400, also called indotecan, represents a new class of non-CPT TOP1i, which is chemically stable, forms persistent TOP1 cleavage complex, and exhibits activity against CPT-resistant cell lines (53–55). Consistent with the results obtained with CPT, DNA fiber analysis indicated that the combination of LMP400 with the BETi, dBET1, significantly impaired the stability of replication forks when compared with treatment with single agents alone or vehicle (DMSO) control (Supplemental Figure 8). We therefore explored the utility of OTX015 in potentiating the antitumor activity of LMP400 in xenograft models (Figure 5A). 22Rv1 and DU145 cells represent CRPC models refractory to many single-agent systemic treatments. Treatment of 22Rv1 xenograft tumors with the BETi OTX015 or the TOP1i LMP400 resulted in a modest reduction in tumor growth, which did not reach statistical significance. The combination treatment of OTX015 and LMP400 synergized and induced a significant reduction in 22Rv1 tumor growth (Figure 5, B and C, and Supplemental Table 5). The single-agent and combination treatments were not associated with noticeable changes in body weight, thereby indicating good tolerability in the animals. Treatment of DU145 xenograft tumors with OTX015 or LMP400 as single agents did not result in any noticeable reduction in tumor growth. In contrast, the combination treatment of OTX015 and LMP400 resulted in a potent reduction in tumor growth (Figure 5, D and E, and Supplemental Table 6). Taken together, these studies present a compelling rationale for combining BETi and TOP1i in the treatment of aggressive cancers.

### TOP1 expression and cancer aggressivity in clinical cohorts.

**Figure 6:**
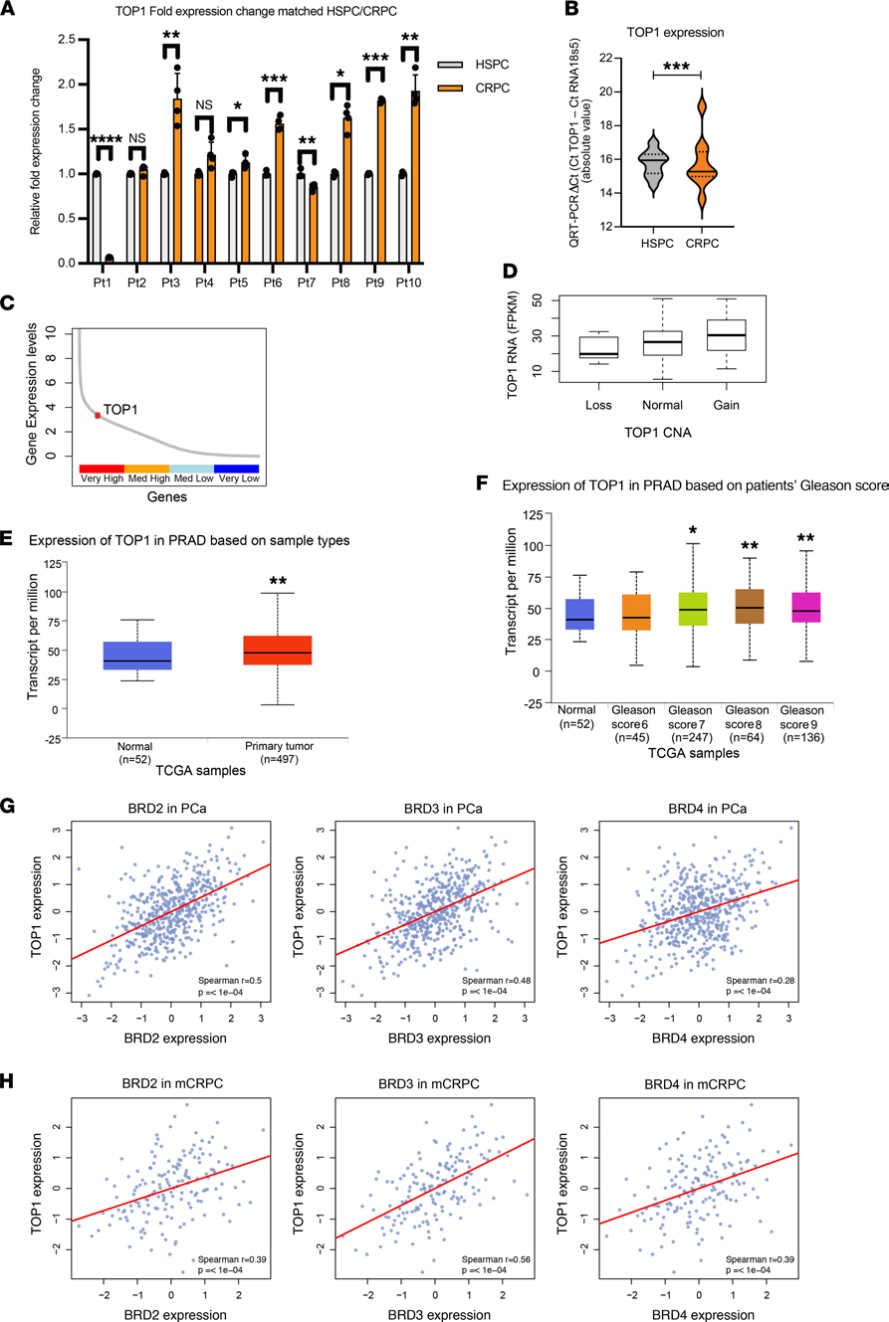
Clinical relevance of *TOP1* transcript abundance and its correlation with *BET* family genes. (**A**) *TOP1* transcript expression changes in 10 individual patients’ matched HSPC and CRPC tissue assessed by quantitative reverse transcription PCR (QRT-PCR). Statistical analysis estimated by multiple paired *t* tests (****P < 0.0001; ***P < 0.001; **P < 0.01; *P < 0.05; error bars, SD of quadruplicates). (**B**) *TOP1* mRNA expression compared with the control gene *RNA 18S ribosomal 5* assessed by QRT-PCR in 10 matched HSPC and CRPC tissue samples. Statistical analysis estimated by 1-sample *t* and Wilcoxon’s test. (**C**) *TOP1* is highly expressed among all expressed genes in mCRPC (Stand Up To Cancer [SU2C] transcriptomic data; *n* = 159). (**D**) *TOP1* expression level versus copy number in mCRPC (SU2C cohort). (**E**) *TOP1* transcript showed a statistically significant increase in primary PCa in comparison with the normal prostate tissue in the TCGA cohort. PRAD, prostate adenocarcinoma. (**F**) *TOP1* transcription stratified by Gleason score. (**G**) TOP1 expression level positively correlates with BRD2, BRD3, and BRD4 in primary PCa (TCGA cohort). (**H**) TOP1 expression level positively correlates with BRD2, BRD3, and BRD4 in mCRPC (SU2C cohort).

To further document the role of TOP1 in drug response, we conducted longitudinal analysis of *TOP1* transcript abundance in patient tumors during PCa progression. Ten patients with matched hormone-sensitive prostate cancer (HSPC) and CRPC biopsies were used to investigate the clinical significance of *TOP1* expression as patients developed CRPC (Figure 6A and Supplemental Table 7). *TOP1* expression was significantly elevated in 6 CRPC biopsies in comparison with matched HSPC. *TOP1* expression was also elevated in 2 additional CRPCs in comparison with matched HSPC, but these were not statistically significantly increased. *TOP1* expression was significantly downregulated in 2 CRPC biopsies in comparison with matched HSPC. Overall, *TOP1* expression increased as patients progressed from HSPC to CRPC (Figure 6B). Consistent with this observation, *TOP1* was highly expressed in mCRPC (Figure 6C). After adjusting for ploidy, 16.7% of mCRPCs exhibited *TOP1* copy number alteration (CNA) gain. *TOP1* CNA was also associated with *TOP1* transcript abundance with near significance (linear regression; *P* = 0.07) (Figure 6D). Analysis of The Cancer Genome Atlas (TCGA) primary PCa data set using the UALCAN portal indicated that *TOP1* expression was significantly elevated in primary PCa in comparison with the normal prostate (Figure 6E) (56). Furthermore, *TOP1* expression was elevated in higher grade PCa (Gleason score 7, 8, and 9) but not in lower grade PCa (Gleason 6) (Figure 6F). We also observed significantly elevated TOP1 protein expression in other cancers, including breast cancer, ovarian cancer, lung adenocarcinoma, and colon cancer (Supplemental Figure 9).

Previously, we reported that BRD4 expression increased significantly as patients with PCa progressed from HSPC to CRPC (18). We next examined the relationship between transcript abundance of *BET* family genes and *TOP1*. Expression of *TOP1* correlated with the expression of *BRD4*, *BRD3*, and *BRD2* in localized PCa as well as mCRPC (Figure 6, G and H). Moreover, this correlation was also observed in the Cancer Cell Line Encyclopedia data set (Supplemental Figure 10). Taken together, these results suggest that the relationship between *BET* family genes and *TOP1* gene expression extends beyond PCa and point to the likely generalizability of this synthetic lethal relationship between BETi and TOP1i.

## Discussion

Men with PCa can be treated with a variety of RT modalities, including external beam radiotherapy and brachytherapy. External beam radiotherapy approaches include both conventionally fractionated regimens (where a small dose of radiation is delivered daily over several weeks) and hypofractionated regimens such as stereotactic body radiation therapy, where high doses of radiation are delivered in fewer fractions under advanced imaging guidance. In general, conventionally fractionated RT outcome is influenced by the repair of DNA damage, redistribution of cells in the cell cycle, repopulation, and reoxygenation of hypoxic tumor areas — collectively called the 4 Rs of radiobiology (57, 58). Treatment with IR predominantly kills actively dividing cells by inducing DNA damage, chromosomal aberrations, transcriptional dysregulation, and mitotic catastrophe, among others. RT preferentially targets tumor cells as these cells replicate at a higher rate in comparison with the adjacent normal cells.

The primary benefit of repeated administration of low-dose radiation is in sparing normal tissue toxicity. For instance, due to its anatomic relation to prostate, rectum is an organ at risk for radiation-related toxicity during prostate irradiation. Repeated administration of low-dose radiation allows normal tissues such as rectum to repair the reversible DNA damage induced by radiation during the intervening periods between radiation treatments. Tumor cells, on the other hand, can have intrinsic DNA repair defects that prevent them from repairing radiation-induced DNA damage as efficiently as surrounding normal tissue. Therefore, repeated low-dose radiation causes repeated DNA damage to tumor cells and induces tumor killing. In addition to tumor cells, endothelial cells have also been reported to be targets of irradiation, and emerging data suggest that multiple mechanisms contribute to the overall success or failure of RT (59–62). Our data indicate that treatment with BETi enhances the efficacy of RT by blocking DNA repair. Given the myriad roles for BET proteins in regulating genome function, we suggest that BETi treatment can also potentiate the effects of RT by additional mechanisms (63).

The success of RT in the treatment of PCa has been substantially enhanced by combination with hormone therapy, which enhances the effects of radiation by impairing repair of radiation-induced DNA damage (64). Although ADT is commonly used to improve the efficacy of RT by blocking DNA repair, the formation of AR-Vs can reverse this effect by promoting DNA repair (43). The combination of ADT and docetaxel in patients with newly diagnosed, high-risk, clinically localized PCa in the neoadjuvant setting is associated with upregulation of both AR and AR-V expression as well as a subset of neuroendocrine and plasticity genes (65). We speculate that the upregulation of AR, AR-Vs, and plasticity genes may partially explain why the combination of RT with adjuvant ADT is associated with improved metastasis-free survival in comparison with the combination of RT with neoadjuvant ADT (66). More generally, there is value in the development of radiosensitizers that are effective regardless of AR or AR-V status. We show that short-term treatment with BETi can enhance the efficacy of radiotherapy and also overcome radioresistance. Importantly, this effect is independent of the androgen signaling axis, though BETi can also regulate AR and AR-V expression. Such short-term treatment approaches are likely to have clinical utility in addressing reversible thrombocytopenia, a commonly observed dose-limiting toxicity with BETi.

We also describe a synthetic lethal relationship between BETi and TOP1i, which is consistent with recent findings demonstrating the regulatory role of BRD4 in regulating TOP1 activity (67). Mechanistically, BETi potentiates the antitumor activity of TOP1i by disrupting replication fork stability. We suggest that BETi can interfere with replication fork stability upon TOP1i treatment by several mechanisms: (A) preventing reversal of stalled replication forks by downregulating RAD51 expression, (B) enhancing conflicts between transcription and DNA replication, and (C) preventing the repair of DNA DSBs arising from collapsed replication forks. All these effects can impinge on the viability of cancer cells. Importantly, BETi-TOP1i combination therapy is likely to be applicable to many cancers.

We suggest that mechanism-guided combination therapies can also address dose-limiting toxicities with BETi. In our study, we used lower doses of BETi, which were well tolerated and not associated with noticeable side effects. For the BETi dose used in the study, single-agent treatment did not show significant reduction in tumor growth. Remarkably, the combination treatments synergized and contributed to a profound reduction in tumor growth. These results indicate that BETi can potentiate the effects of diverse therapeutic agents that damage DNA, ranging from localized RT to systemic targeted therapies. Such combinatorial therapies have utility in the radical treatment of both localized and metastatic PCa.

## Methods

### Cell culture and transfection.

Further information can be found in Supplemental Methods. Cell lines LNCaP, VCaP, 22Rv1, DU145, and U2OS were obtained from the American Type Culture Collection. LNCaP, DU145, and 22Rv1 were maintained in RPMI-1640 medium (Thermo Fisher Scientific) containing 10% fetal bovine serum (FBS) in a 5% CO_2_ humidified incubator. U2OS were cultured in McCoy’s 5A medium (Thermo Fisher Scientific) and supplemented with 10% FBS. HEK293T (see Supplemental Methods) and VCaP were maintained in DMEM plus 10% FBS. All cell lines were verified via genotyping and tested negative throughout this project for mycoplasma contamination by MycoAlert Mycoplasma Detection Kit (Lonza).

Nontargeting siRNA, (D-001810-10-50. Sequences, UGGUUUACAUGUCGACUAA, UGGUUUACAUGUUGUGUGA,UGGUUUACAUGUUUUCUGA, UGGUUUACAUGUUUUCCUA), siRAD51 (L-003530-00-0005), siBRCA1 (L-003461-00-0005), siBRD2 (set of 4) (J-004937-06 CACGAAAGCUACAGGAUGU, J-004937-07 GGGCCGAGUUGUGCAUAUA, J-004937-08 CCUAAGAAGUCCAAGAAAG, J-004937-09 GUCCUUUCCUGCCUACGUA), siBRD3 (set of 4) (J-004936-05 AAUUGAACCUGCCGGAUUA, J-004936-06 CGGCUGAUGUUCUCGAAUU, J-004936-07 GGAGAGAUAUGUCAAGUCU, J-004936-08 GCGAAUGUAUGCAGGACUU), and siBRD4 (set of 4) (J-004937-06 AAACCGAGAUCAUGAUAGU, J-004937-07 CUACACGACUACUGUGACA, J-004937-08 AAACACAACUCAAGCAUCG, J-004937-09 CAGCGAAGACUCCGAAACA) were purchased from Dharmacon/Horizon Discovery. Each siRNA was transfected into target cells using Lipofectamine RNAiMAX Reagent (13778150, Invitrogen, Thermo Fisher Scientific) according to the manufacturer’s protocol.

RAD51 ORF clone (clone ID: OHu15695D) and Flag-BRD4 (clone ID: OHu15695D) were purchased from GenScript. Plasmids were transfected using Lipofectamine 3000 Transfection Reagent (L3000015, Invitrogen, Thermo Fisher Scientific) according to the manufacturer’s protocol.

### PDE studies.

Excised tissue samples were processed and cultured ex vivo as previously described (68). Briefly, tissue samples were incubated on gelatin sponges in RPMI-1640 culture medium containing 10% FBS, 0.01 mg/mL insulin, and 0.01 mg/mL hydrocortisone. Explants were treated with JQ1 (Selleckchem) (1 μM) 1 day prior to IR (2 Gy) treatment. PDEs were analyzed 1 hour and 8 hours after IR treatment. Representative tissues were fixed in 10% formalin at 4°C overnight and subsequently processed into paraffin blocks. Sections were stained with H&E and examined to confirm and quantify the presence/proportion of tumor cells. Pan-Cytokeratin analysis in PDEs was done using pan-Cytokeratin antibody (catalog GTX29377, GeneTex) using a previously described protocol (42).

### Mice and xenografts.

Athymic nude male mice (4 weeks old) were obtained from Charles River (strain 490). Cultured human prostate cancer cells DU145 (2 × 10^6^ cells per animal) or 22Rv1 (3 × 10^6^ cells per animal) were first mixed with 50% Corning Matrigel Membrane Matrix (08-774-391, Thermo Fisher Scientific), then injected subcutaneously in the dorsum of the mouse’s neck following published guidelines (69). The treatments were initiated after the tumors reached a volume of 110–150 mm^3^. X-RAD 225Cx irradiator (Precision X-Ray Inc.) was used to administer localized fractionated IR to mice. OTX015 (100 mg/kg; M2903, Abmole Bioscience) was given via oral gavage once a day. OTX015 was dissolved in 1 part 100% ethanol and 9 parts vehicle (0.5% methylcellulose plus 0.1% Tween 80 from MilliporeSigma in double-distilled water), followed by ultrasonication for about 20 minutes at 4°C. LMP400 (10 mg/kg) was administered via IP injection once a day. LMP400 was dissolved in 1 part 20 mM HCl/10 mM citric acid and 9 parts 5% dextrose water. LMP400 was obtained from the NIH. BD Ultra-Fine short insulin syringes (BD328438, Becton, Dickinson and Company) were used for tumor inoculation and IP injections. Tumor volume was measured 2 to 3 times per week using Mitutoyo Absolute 500-196-20 Digital Caliper 0-6. Mouse body weight was measured 2 to 3 times per week using a portable scale (Braintree Scientific, CB-1001).

### Statistics.

Mice were first mixed among cages and then assigned randomly to treatment groups for all in vivo studies. *P* values for γ-H2AX foci and RAD51 foci staining were obtained using 2-tailed Student’s *t* test. *P* values for in vivo data were obtained using 2-tailed Student’s *t* test. *P* values for DNA fiber assay were assessed using 2-tailed Mann-Whitney *U* test. *P* values for flow cytometry data were assessed using 2-tailed Student’s *t* test. The *P* values were adjusted by Benjamini-Hochberg procedure for multiple comparisons. *P* values that are considered significant are as follows: ****P < 0.0001, ***P < 0.001; **P < 0.01, *P < 0.05.

### Study approval.

All animal experimental procedures were approved by the UT Southwestern Institutional Animal Care and Use Committee, Dallas, Texas, USA. Patients at UT Southwestern provided written informed consent allowing the use of discarded surgical samples for research purposes according to an institutional review board–approved protocol. Deidentified patient tumors were obtained from the UT Southwestern Tissue Management Shared Resource after institutional review board (Dallas, Texas, USA) approval (STU-032011–187). Patients were identified from a population of men with CRPC treated at the Royal Marsden NHS Foundation Trust. All patients had given written informed consent and were enrolled in institutional protocols approved by the Royal Marsden NHS Foundation Trust Hospital (London, United Kingdom) ethics review committee (reference 04/Q0801/60). Human biological samples were sourced ethically, and their research use was in accord with the terms of the informed consent provided.

## Author contributions

RSM and XL conceived and designed the study; XL, GB, SC, SM, MH, GVR, JDB, and RSM developed methodology; XL, GB, SC, SM, MH, SL, CB, and MDLDFDLM acquired data; XL, GB, SC, WY, SM, MH, SL, YG, CB, BPCC, GVR, JDB, YP, and RSM analyzed and interpreted data; RSM and XL wrote, reviewed, and revised the manuscript with input from all authors; PGA, SB, and YP provided administrative, technical, or material support; and RSM provided study supervision.

## Supplementary Material

Supplemental data

## Version 1 03/29/2022

In-Press Preview

## Version 2 05/09/2022

Electronic publication
