# Supplementary material for: Targeting radioresistance and replication fork stability in prostate cancer
Source: JCI Insight. 2022 May 9;7(9):e152955. doi: 10.1172/jci.insight.152955 (PMC9090241; doi:10.1172/jci.insight.152955)
Supplement: Supplemental data [file jciinsight-7-152955-s030.pdf]

Supplementary figure legends

Figure S1. The effect of BETi treatment on the repair of DNA DSBs.

(A) Images of patient-derived explants (PDEs). PCa tissue pieces were incubated on gelatin sponge in RPMI-1640 culture medium containing 10% FBS, 0.01 mg/ml insulin and 0.01 mg/ml hydrocortisone. **(B)** H&E staining of a representative PDE showed PCa morphology. **(C)** The epithelial marker pan Cytokeratin (red, 40X) immunofluorescence staining of a representative PDE. Blue is DAPI. Scale bar, 50 µm. **(D)** The effect of OTX015 treatment on NHEJ DNA repair pathway. NHEJ flow cytometry assay was conducted with OTX015 at indicated dosages in engineered HEK293T cells. Unpaired two-tailed Student's t-test was applied (***P < 0.001; **P < 0.01; *P < 0.05; Error bars, SD of three technical replicates).

Figure S2. The role of BET proteins and the effect of BETi treatment on HR DNA repair pathway.

(A) HR assay schematic. Engineered HEK293 DR-GFP cells bearing fragments of GFP was transfected with plasmids harboring I-SceI. Repair of I-SceI induced DNA DSBs via HR pathway results in the expression of full GFP protein, which can be detected by flow cytometry. The percentage of GFP positive cells following I-SceI transfection directly correlates with HR activity in this assay.
(B) Representative flow cytometry images to describe the effect of JQ1 on HR DNA repair. **(C)** HR flow cytometry assay was conducted with JQ1 at indicated dosages. Error bars, SD of three technical replicates. **(D)** HR flow cytometry assay was conducted with OTX015 at displayed dosages. Error bars, SD of three technical replicates. **(E)** HR pathway activity was measured via flow cytometry in cells treated with the indicated siRNAs. siBRCA1 and siRAD51 were used as positive controls. Error bars, SD of three technical replicates. **(F)** siRNA knockdown validation by QRT-PCR. Error bars, SD of three technical replicates. **(E-F)** (p) indicates pooled siRNA. siRNA (1), (2), (3) or (4) represent the individual siRNA. Unpaired two-tailed Student's t-test was applied (***P < 0.001; **P < 0.01; *P < 0.05 for all panels).

Figure S3. The role of BET proteins and the effect of BETi treatment on HR DNA repair pathway in PCa cell lines.

(A) HR DNA repair pathway activity was analyzed using HR QPCR assay in PCa cell lines LNCaP, VCaP, 22Rv1 and DU145 upon BETi treatment. **(B)** HR DNA repair pathway activity was analyzed using HR QPCR assay in PCa cell lines LNCaP, VCaP, 22Rv1 and DU145 upon various siRNA treatments. **(C)** siRNA knockdown validation. Unpaired two-tailed Student's t-test was applied for all panels (***P < 0.001; **P < 0.01; *P < 0.5; Error bars, SD of three technical replicates for all panels).

Figure S4. The role of BET proteins in IR-induced RAD51 foci formation.

(A) Representative images of RAD51 nuclear foci in mock-irradiated or gamma-irradiated U2OS cells. Cells were treated with JQ1 (1 μM or 10 μM) for 24 hrs prior to IR treatment. Cells were immunostained with an anti-RAD51 antibody (red) and the nuclei were stained with DAPI (blue). Cells were analyzed 4 hours post IR treatment. Scale bar = 20 μm. **(B)** Quantification of RAD51 foci in U2OS cells treated with JQ1 for 24 hrs prior to IR. Cells were analyzed 4 hrs post IR-treatment. **(C)** Quantification of RAD51 foci in U2OS cells that were simultaneously co-treated with JQ1 and IR**.** Cells were analyzed 4 hrs post JQ1+IR treatment. **(D)** Quantification of RAD51 foci in U2OS cells that were treated by dBET1 24 hrs prior to IR. Cells were analyzed 4 hrs post IR-treatment. **(E)** The effect of BETi on BET protein level in U2OS whole cell lysate (left) or chromatin fraction (right). **(F)** The effect of BRD4, BRD3 and/or BRD2 knockdown on IR-induced RAD51 foci formation in U2OS cells. **(G)** The effect of BRD4 overexpression on IR-induced RAD51 foci formation in U2OS cells. Western blot validates the overexpression of either flag protein or BRD4 protein. **(H)**The effect of JQ1 treatment on RAD51 protein expression in LNCaP cells. **(I)** Integrated genome view of *RAD51* gene in VCaP cells. Each row represents Chip-Seq data with the indicated antibodies and treatment. Unpaired two-tailed Student's t-test (***P < 0.001, **P < 0.01) was applied in **(B, C, D, F & G).**

Figure S5. Over-expression of RAD51 promotes HR activity.

(A) Overexpression of RAD51 in U2OS cells was verified by QPCR. ***P < 0.001, Student's t-test. **(B)** Representative images for RAD51 foci analysis in U2OS cells upon RAD51 over-expression with or without irradiation (IR 6 Gy). pcDNA3.1 was used as a mock control. Cells were analyzed 4 hrs post IR treatment. **(C)** Homologous Recombination (HR) activity was analyzed upon RAD51 over-expression in LNCaP, VCaP, 22Rv1 and DU145 PCa cells. ***P < 0.001, **P < 0.01, Student's t-test. **(D)** Over-expression of RAD51 in the four cell lines was verified by QPCR. ***P < 0.001, **P < 0.01, Student's t-test.

Figure S6. BETi treatment results in the transcriptional down-regulation of DNA repair genes.

(A-E) Relative transcript expression of the indicated DNA repair genes upon JQ1 treatment was analyzed by QPCR in LNCaP, VCaP, 22Rv1 and DU145 PCa cells. ***P < 0.001, **P < 0.01, *P < 0.05, Student's t-test (top panel). Integrated genome view of the indicated genes in VCaP cells. Each row represents RNA Pol II Chip-Seq data with the indicated treatment.

Figure S7. Combination Index (CI) of JQ1 and CPT in PCa cell lines.

The effect of JQ1 + CPT co-treatment in the four PCa cell lines was tested. CI > 1 indicates antagonism; CI = 1 indicates additive effect; CI < 1 indicates synergism.

Figure S8. DNA fiber assay with dBET1 and LMP400 in LNCaP cells.

(A) Labeling scheme. Cells were pulse-labeled first with IdU (100 μM, 30 min) and then with CldU (100 μM, 30 min), followed by drug treatment for 5 hrs. **(B)** The effect of treatment with dBET1 and/or LMP400 on DNA replication fork stability. The length of labeled DNA tracks, IdU (red) and CldU (green), was scored and displayed in scatter dot plots (left panel). CldU/IdU ratio of each track was calculated and displayed in box-&-whisker plots (right panel). Boxes: 25th – 75th percentile; Whiskers: 10th – 90th percentile. Two-tailed Mann-Whitney U test was applied (****P < 0.0001; ***P < 0.001; **P < 0.01; *P < 0.05; n.s., non-significant).

Figure S9. Expression of TOP1 protein in various cancers.

TOP1 protein expression was analyzed in breast cancer, ovarian cancer, lung adenocarcinoma and colon cancer. Box plots were adapted from UALCAN (Two-tailed Student's t-test; ***P < 0.0001).

Figure S10. Correlation analysis between *BRD2/3/4* transcript expression and *TOP1* transcript expression in cancer cell lines.

BRD2/3/4 transcript expression was compared to *TOP1* transcript expression using the Cancer Cell Line Encyclopedia (CCLE) RNA-Seq database.

Figure S1

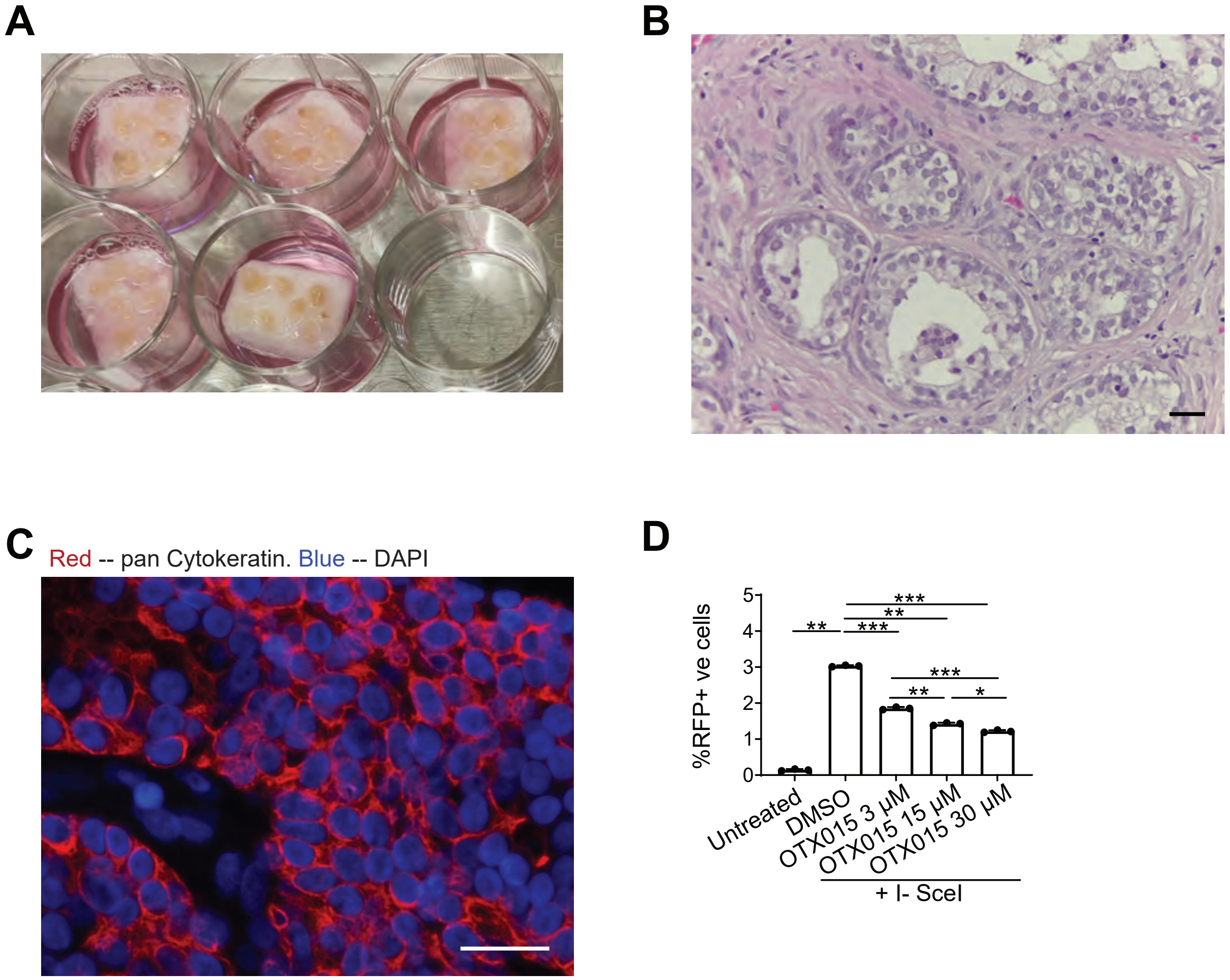
A
B
C
Red -- pan Cytokeratin. Blue -- DAPI
D
%RFP+ ve cells
0
1
2
3
4
5
**
***
**
***
***
**
*
Untreated
DMSO
OTX015 3 μM
OTX015 15 μM
OTX015 30 μM
+ I- SceI

Figure S2

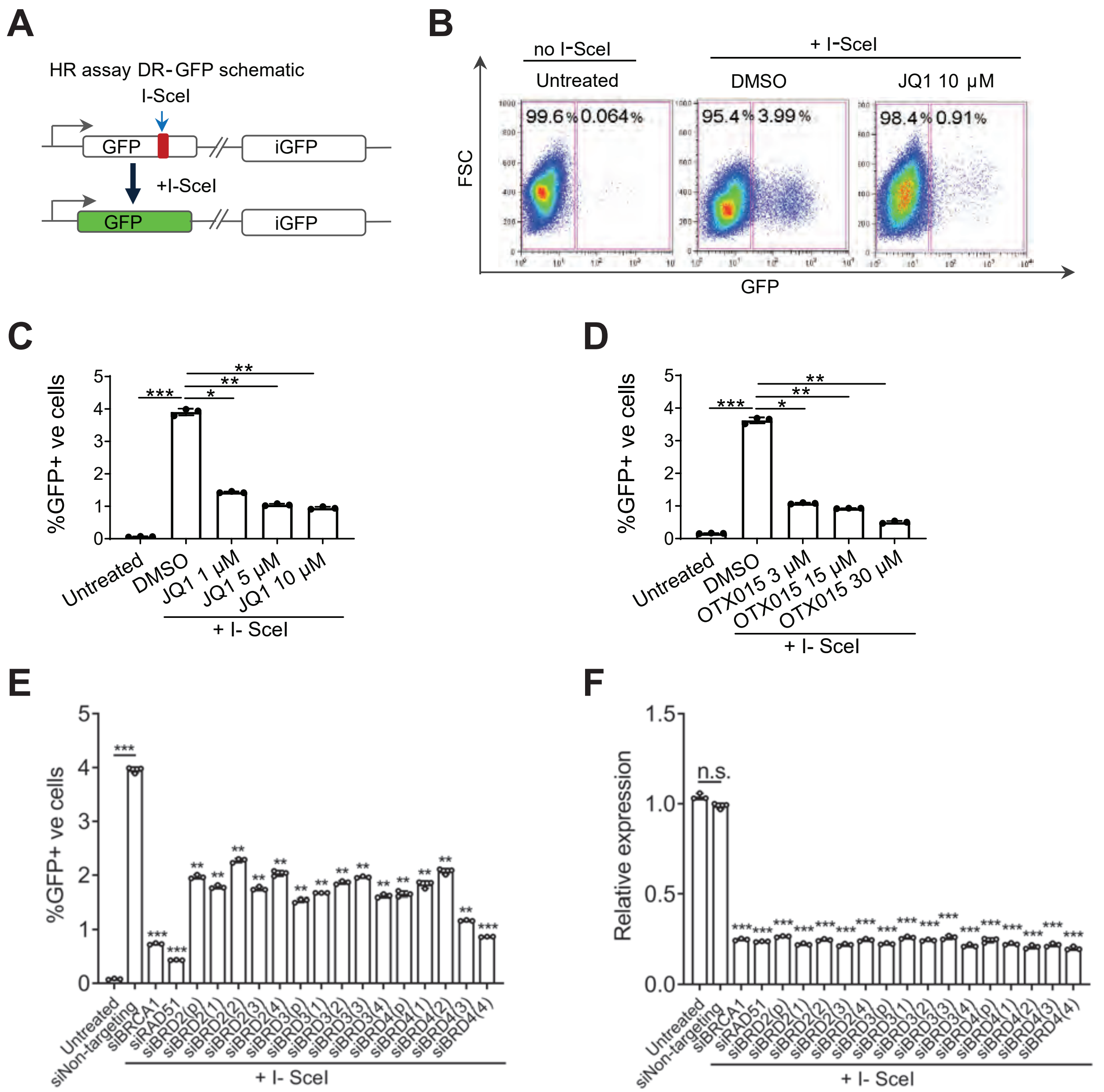

A
HR assay DR-GFP schematic
I-SceI
GFP
iGFP
+I-SceI
GFP
iGFP
B
no I-SceI
+ I-SceI
Untreated
DMSO
JQ1 10 μM
99.6% 0.064%
95.4% 3.99%
98.4% 0.91%
FSC
GFP
C
%GFP+ ve cells
Untreated
DMSO
JQ1 1 μM
JQ1 5 μM
JQ1 10 μM
+ I- SceI
D
%GFP+ ve cells
Untreated
DMSO
OTX015 3 μM
OTX015 15 μM
OTX015 30 μM
+ I- SceI
E
%GFP+ ve cells
Untreated
siNon-targeting
siBRCA1
siRAD51
siBRD2(p)
siBRD2(1)
siBRD2(2)
siBRD2(3)
siBRD2(4)
siBRD3(p)
siBRD3(1)
siBRD3(2)
siBRD3(3)
siBRD3(4)
siBRD4(p)
siBRD4(1)
siBRD4(2)
siBRD4(3)
siBRD4(4)
+ I- SceI
F
Relative expression
n.s.
Untreated
siNon-targeting
siBRCA1
siRAD51
siBRD2(p)
siBRD2(1)
siBRD2(2)
siBRD2(3)
siBRD2(4)
siBRD3(p)
siBRD3(1)
siBRD3(2)
siBRD3(3)
siBRD3(4)
siBRD4(p)
siBRD4(1)
siBRD4(2)
siBRD4(3)
siBRD4(4)
+ I- SceI

Figure S3

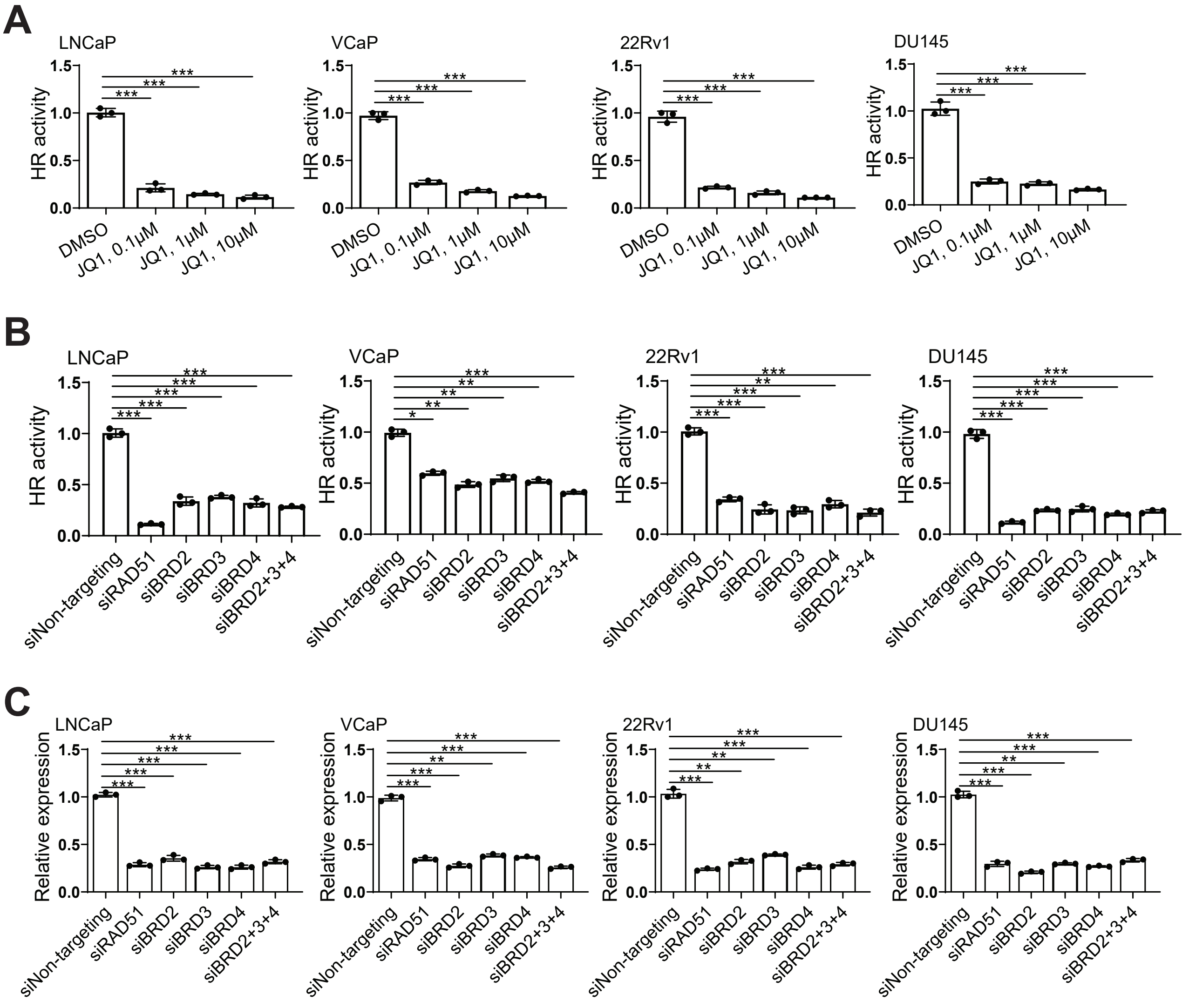
A
LNCaP
VCaP
22Rv1
DU145
HR activity
DMSO
JQ1, 0.1μM
JQ1, 1μM
JQ1, 10μM
B
siNon-targeting
siRAD51
siBRD2
siBRD3
siBRD4
siBRD2+3+4
C
Relative expression

Figure S4

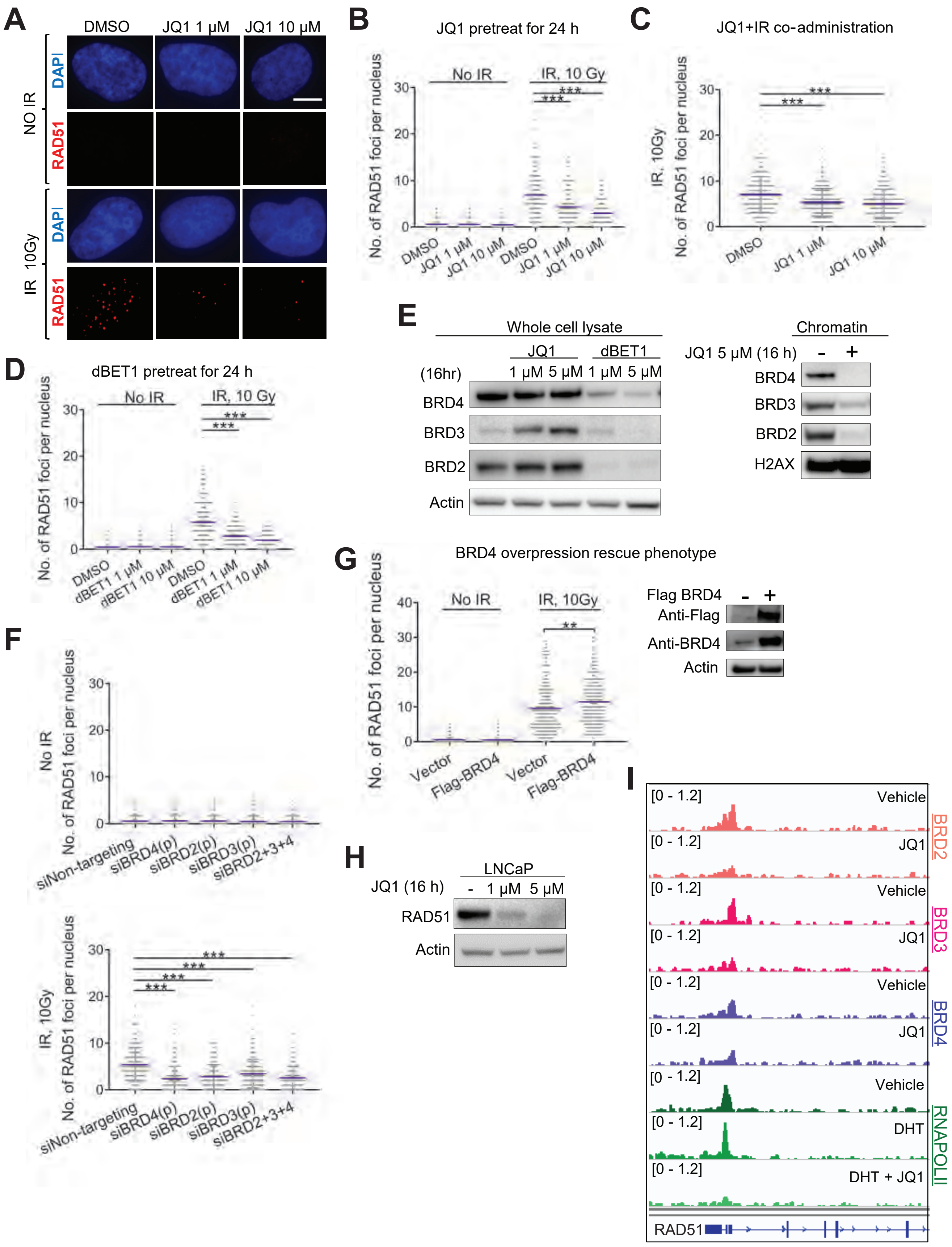

A
DMSO
JQ1 1 μM
JQ1 10 μM
NO IR
DAPI
RAD51
IR 10Gy
B
JQ1 pretreat for 24 h
No IR
IR, 10 Gy
No. of RAD51 foci per nucleus
DMSO
JQ1 1 μM
JQ1 10 μM
C
JQ1+IR co-administration
IR, 10Gy
D
dBET1 pretreat for 24 h
dBET1 1 μM
dBET1 10 μM
E
Whole cell lysate
JQ1
dBET1
(16hr)
1 μM 5 μM 1 μM 5 μM
BRD4
BRD3
BRD2
Actin
Chromatin
JQ1 5 μM (16 h)
H2AX
F
siNon-targeting
siBRD4(p)
siBRD2(p)
siBRD3(p)
siBRD2+3+4
G
BRD4 overpression rescue phenotype
No IR
IR, 10Gy
Vector
Flag-BRD4
Flag BRD4
Anti-Flag
Anti-BRD4
H
LNCaP
JQ1 (16 h)
1 μM 5 μM
RAD51
I
[0 - 1.2]
Vehicle
JQ1
DHT
DHT + JQ1
BRD2
BRD3
BRD4
RNAPOLII
RAD51

Figure S5

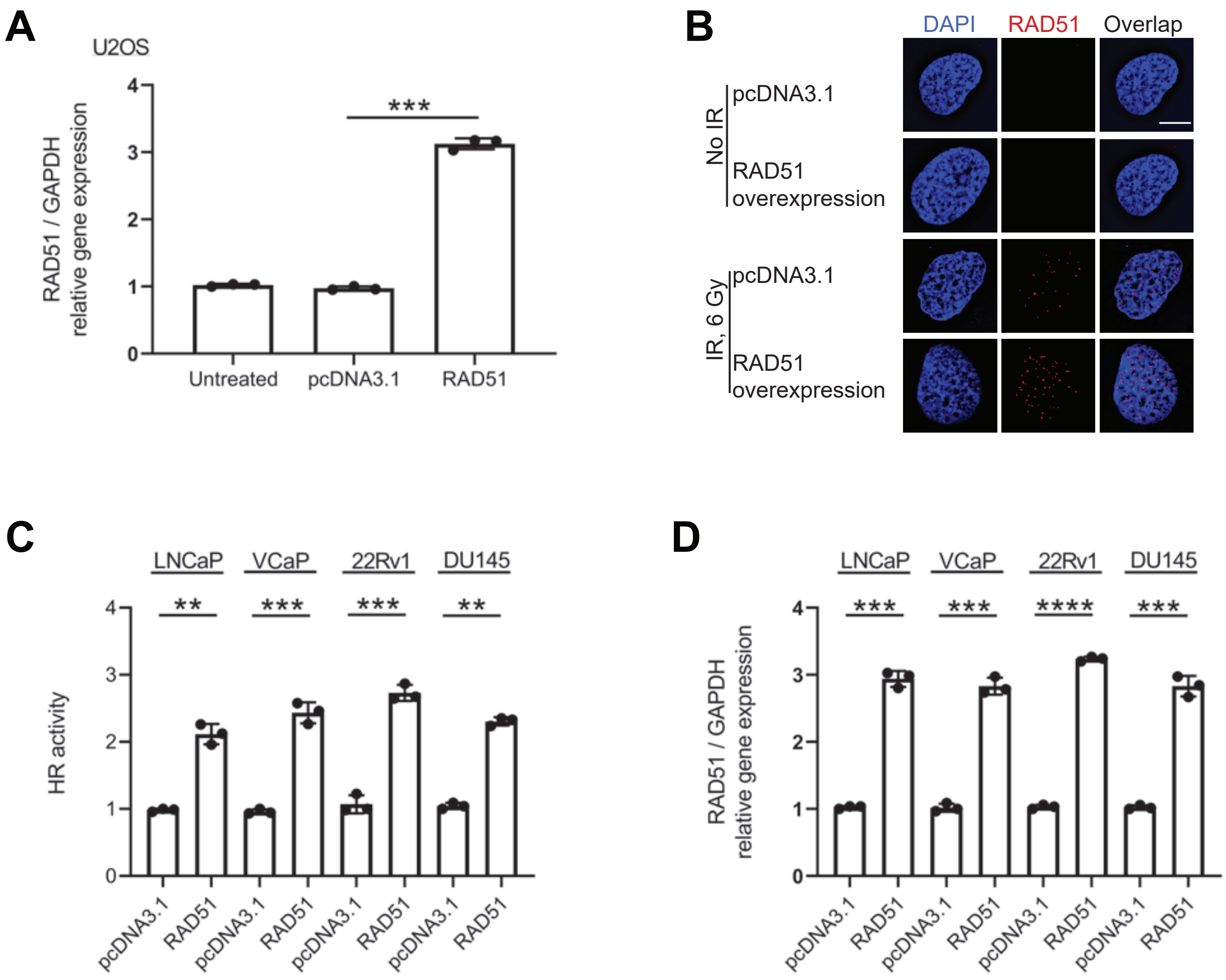

A
U2OS
RAD51 / GAPDH relative gene expression
***
0
1
2
3
4
Untreated
pcDNA3.1
RAD51
B
DAPI
RAD51
Overlap
No IR
pcDNA3.1
RAD51 overexpression
IR, 6 Gy
pcDNA3.1
RAD51 overexpression
C
LNCaP
VCaP
22Rv1
DU145
**
***
***
**
HR activity
pcDNA3.1
RAD51
D
LNCaP
VCaP
22Rv1
DU145
***
***
****
***
RAD51 / GAPDH relative gene expression
pcDNA3.1
RAD51

Figure S6

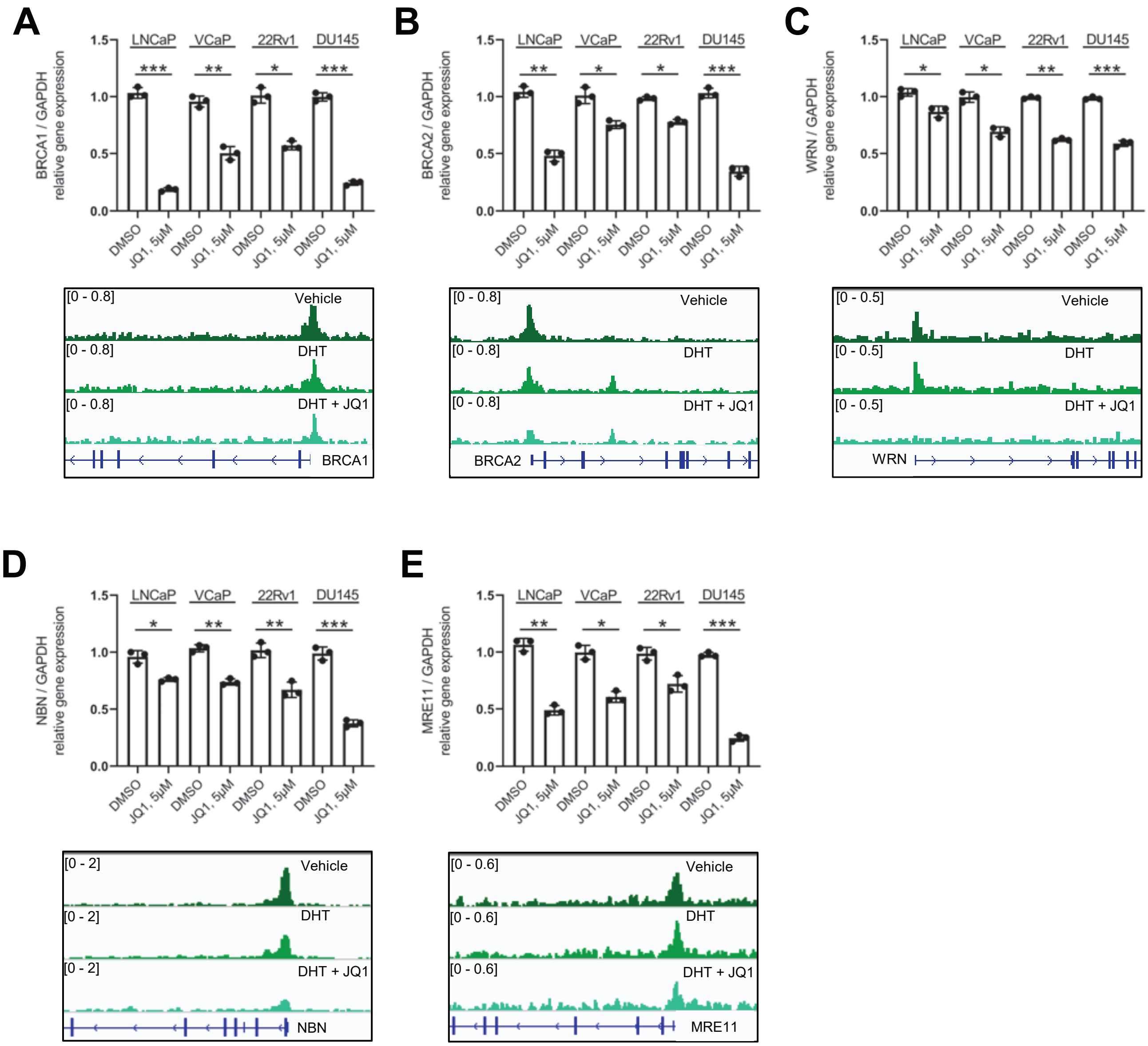

A
B
C
D
E
BRCA1 / GAPDH relative gene expression
BRCA2 / GAPDH relative gene expression
WRN / GAPDH relative gene expression
NBN / GAPDH relative gene expression
MRE11 / GAPDH relative gene expression
LNCaP
VCaP
22Rv1
DU145
DMSO
JQ1, 5μM
[0 - 0.8]
[0 - 0.5]
[0 - 2]
[0 - 0.6]
Vehicle
DHT
DHT + JQ1
BRCA1
BRCA2
WRN
NBN
MRE11

Figure S7

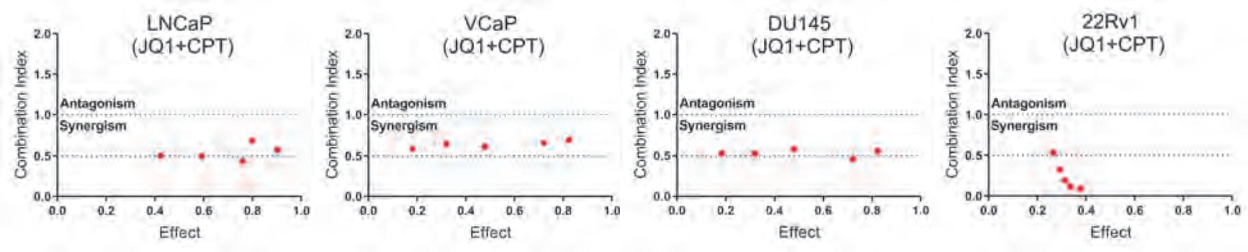
LNCaP
(JQ1+CPT)
VCaP
(JQ1+CPT)
DU145
(JQ1+CPT)
22Rv1
(JQ1+CPT)
Combination Index
Antagonism
Synergism
Effect
0.0
0.5
1.0
1.5
2.0
0.2
0.4
0.6
0.8

Figure S8

A

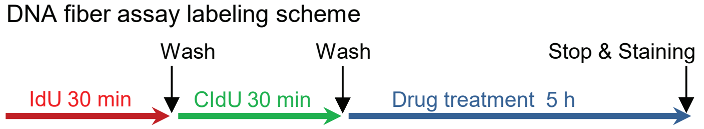
DNA fiber assay labeling scheme
Wash
Wash
Stop & Staining
IdU 30 min
CldU 30 min
Drug treatment 5 h

B

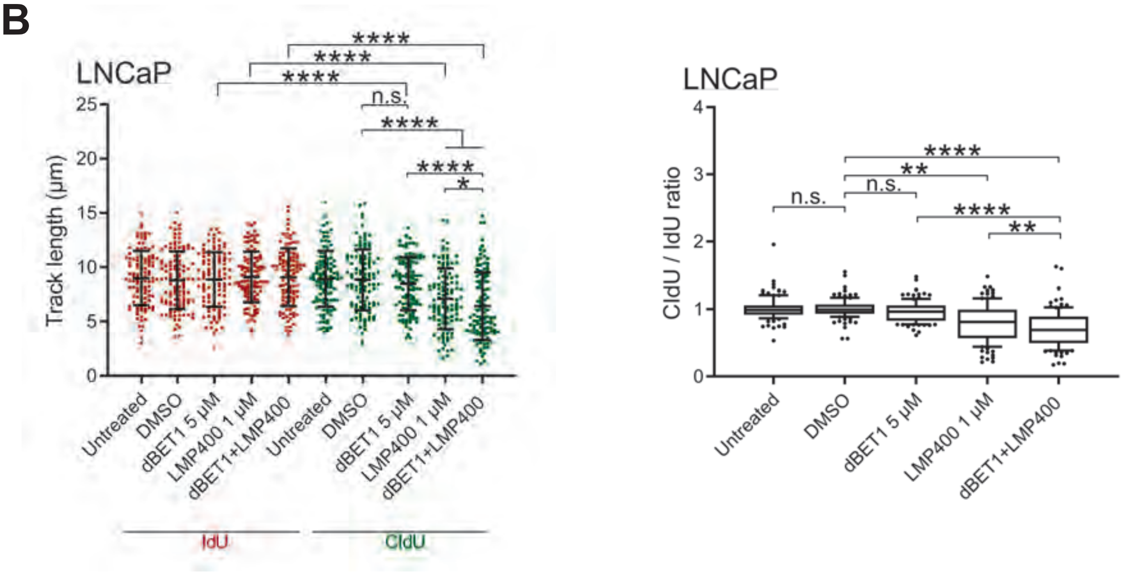
LNCaP
Track length (µm)
Untreated
DMSO
dBET1 5 µM
LMP400 1 µM
dBET1+LMP400
IdU
CldU
n.s.
LNCaP
CldU / IdU ratio

Figure S9

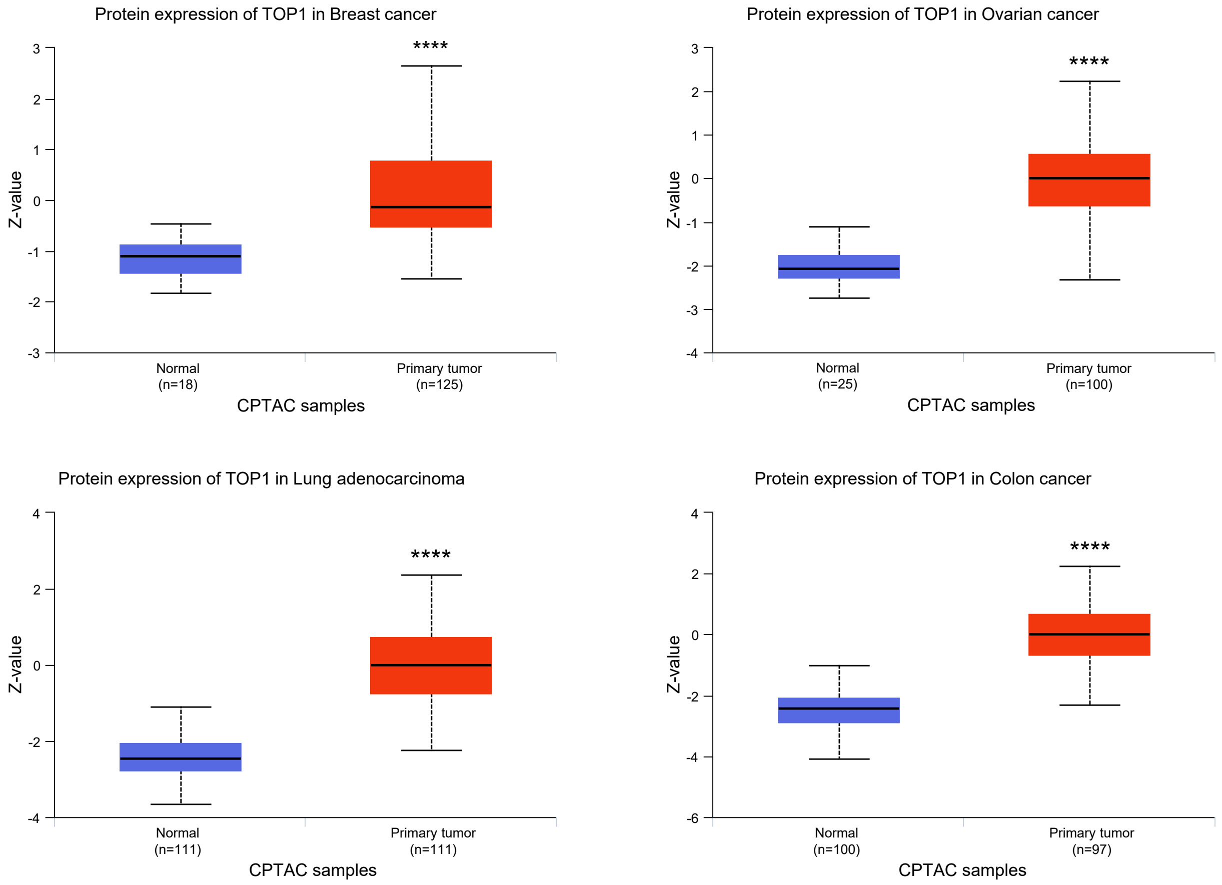

Protein expression of TOP1 in Breast cancer
Z-value
3
2
1
0
-1
-2
-3
****
Normal
(n=18)
Primary tumor
(n=125)
CPTAC samples
Protein expression of TOP1 in Ovarian cancer
Z-value
3
2
1
0
-1
-2
-3
-4
****
Normal
(n=25)
Primary tumor
(n=100)
CPTAC samples
Protein expression of TOP1 in Lung adenocarcinoma
Z-value
4
2
0
-2
-4
****
Normal
(n=111)
Primary tumor
(n=111)
CPTAC samples
Protein expression of TOP1 in Colon cancer
Z-value
4
2
0
-2
-4
-6
****
Normal
(n=100)
Primary tumor
(n=97)
CPTAC samples

Figure S10

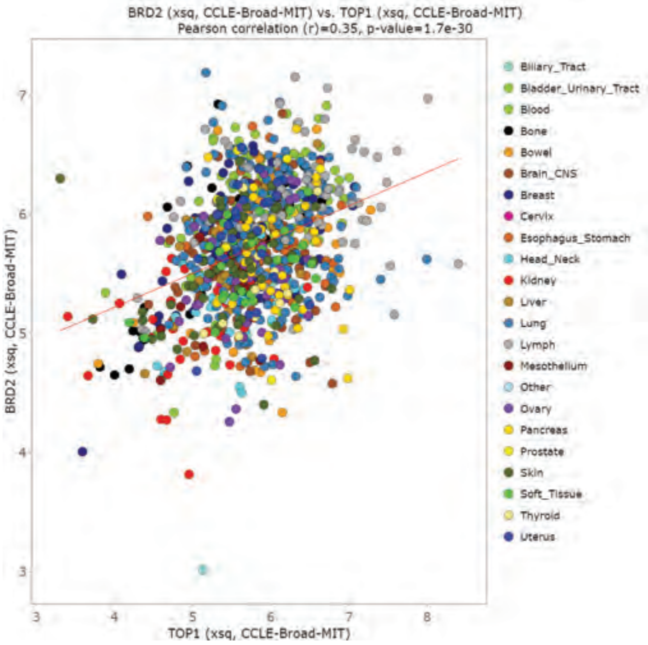

BRD2 (xsq, CCLE-Broad-MIT) vs. TOP1 (xsq, CCLE-Broad-MIT)
Pearson correlation (r)=0.35, p-value=1.7e-30
BRD2 (xsq, CCLE-Broad-MIT)
TOP1 (xsq, CCLE-Broad-MIT)
Biliary_Tract
Bladder_Urinary_Tract
Blood
Bone
Bowel
Brain_CNS
Breast
Cervix
Esophagus_Stomach
Head_Neck
Kidney
Liver
Lung
Lymph
Mesothelium
Other
Ovary
Pancreas
Prostate
Skin
Soft_Tissue
Thyroid
Uterus

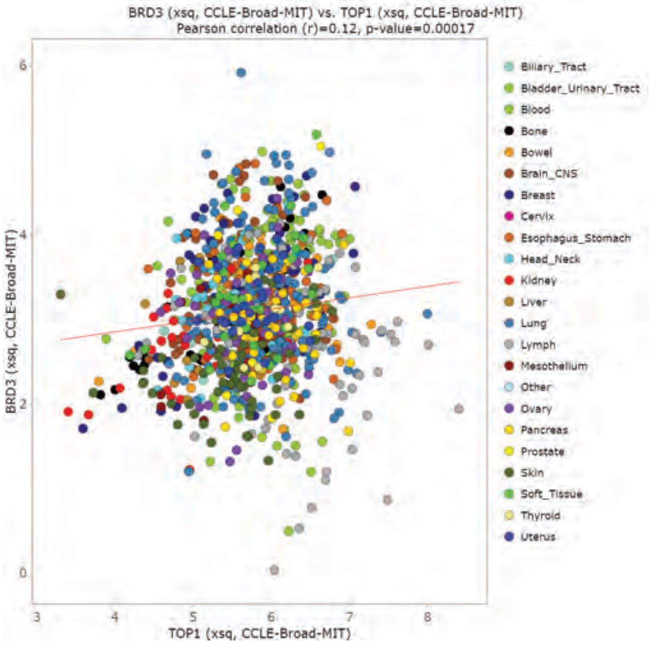

BRD3 (xsq, CCLE-Broad-MIT) vs. TOP1 (xsq, CCLE-Broad-MIT)
Pearson correlation (r)=0.12, p-value=0.00017
BRD3 (xsq, CCLE-Broad-MIT)
TOP1 (xsq, CCLE-Broad-MIT)
Biliary_Tract
Bladder_Urinary_Tract
Blood
Bone
Bowel
Brain_CNS
Breast
Cervix
Esophagus_Stomach
Head_Neck
Kidney
Liver
Lung
Lymph
Mesothelium
Other
Ovary
Pancreas
Prostate
Skin
Soft_Tissue
Thyroid
Uterus

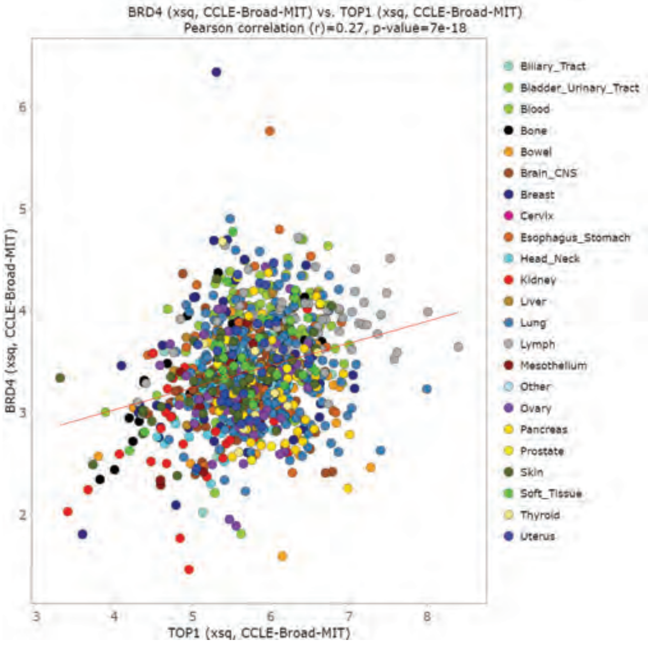

BRD4 (xsq, CCLE-Broad-MIT) vs. TOP1 (xsq, CCLE-Broad-MIT)
Pearson correlation (r)=0.27, p-value=7e-18
BRD4 (xsq, CCLE-Broad-MIT)
TOP1 (xsq, CCLE-Broad-MIT)
Biliary_Tract
Bladder_Urinary_Tract
Blood
Bone
Bowel
Brain_CNS
Breast
Cervix
Esophagus_Stomach
Head_Neck
Kidney
Liver
Lung
Lymph
Mesothelium
Other
Ovary
Pancreas
Prostate
Skin
Soft_Tissue
Thyroid
Uterus

Supplemental Tables

Table S1. Related to Figure 1A-B. Baseline characteristics of prostate cancer patients whose primary prostate cancer explants were analyzed.

PCa explant #	Gleason Score	PSA (ng/mL)	Grade group	Prior treatment
**1**	7 (3+4)	7.22	2	No
**2**	7 (4+3)	9.38	3	No
**3**	7 (4+3)	7.38	3	No
**4**	7 (3+4)	7.2	2	No
**5**	9 (4+5)	5.7	5	Yes
**6**	7 (3+4)	7.4	2	No
**7**	7 (4+3)	5.23	3	No
**8**	7 (3+4)	4.11	2	No
**9**	7 (4+3)	8	3	No
**10**	7 (4+3)	13.45	3	No

Table S2. P-values for Figure 1D

Treatment arms	Tumor volume, FDR adjusted P-value (Two-tailed Student's t-test)
Vehicle vs. RT	P = 0.030754
Vehicle vs. OTX015	P = 0.940579
Vehicle vs. RT+OTX015	P < 0.000025
RT vs. RT+OTX015	P = 0.000102
OTX015 vs. RT+OTX015	P < 0.000025

Table S3. P-values for Figure 2B

Treatment arms	Tumor volume, FDR adjusted P-value (Two-tailed Student's t-test)
Vehicle vs. RT	P = 0.904715
Vehicle vs. OTX015	P = 0.904715
Vehicle vs. RT+OTX015	P = 0.000055
RT vs. RT+OTX015	P < 0.000050
OTX015 vs. RT+OTX015	P = 0.000208

Table S4. P-values for Figure 4G

LNCaP<br>Cell Proliferation assay	**FDR adjusted P-value**<br>**(Two-tailed Student's t-test)**
DMSO vs. JQ1	P = 0.00052
DMSO vs. CPT	P < 0.0000125
DMSO vs. JQ1+CPT	P < 0.0000125
JQ1 vs. JQ1+CPT	P < 0.0000125
CPT vs. JQ1+CPT	P < 0.0000125

**VCaP**<br>Cell Proliferation assay	**FDR adjusted P-value**<br>**(Two-tailed Student's t-test)**
DMSO vs. JQ1	P = 0.070175
DMSO vs. CPT	P = 0.515700
DMSO vs. JQ1+CPT	P < 0.000017
JQ1 vs. JQ1+CPT	P < 0.000017
CPT vs. JQ1+CPT	P < 0.000017

**22Rv1**<br>Cell Proliferation assay	**FDR adjusted P-value**<br>**(Two-tailed Student's t-test)**
DMSO vs. JQ1	P = 0.001633
DMSO vs. CPT	P = 0.003740
DMSO vs. JQ1+CPT	P < 0.000050
JQ1 vs. JQ1+CPT	P = 0.001750
CPT vs. JQ1+CPT	P = 0.000950

**DU145**<br>Cell Proliferation assay	**FDR adjusted P-value**<br>**(Two-tailed Student's t-test)**
DMSO vs. JQ1	P = 0.34722
DMSO vs. CPT	P = 0.34722
DMSO vs. JQ1+CPT	P = 0.03070
JQ1 vs. JQ1+CPT	P = 0.07230
CPT vs. JQ1+CPT	P = 0.07230

Table S5. P-values for Figure 5B

Treatment arms	Tumor volume, FDR adjusted P-value (Two-tailed Student's t-test)
Vehicle vs. LMP400	P = 0.890305
Vehicle vs. OTX015	P = 0.174080
Vehicle vs. LMP400+OTX015	P < 0.000025
LMP400 vs. LMP400+OTX015	P < 0.000025
OTX015 vs. LMP400+OTX015	P = 0.006955

Table S6. P-values for Figure 5D

Treatment arms	Tumor volume, FDR adjusted P-value (Two-tailed Student's t-test)
Vehicle vs. LMP400	P = 0.231103
Vehicle vs. OTX015	P = 0.231103
Vehicle vs. LMP400+OTX015	P = 0.000415
LMP400 vs. LMP400+OTX015	P < 0.000050
OTX015 vs. LMP400+OTX015	P = 0.002507

Table S7. Related to Figures 6A and 6B. Baseline characteristics for those patients.

	Age at diagnosis	Histology (Adeno-carcinoma 1, other 2)	Gleason (1: < or = 7, 2 > 7)	PSA at diagnosis	T at diagnosis (1,2,3,4, U)	N at diagnosis 0,1, U	M at diagnosis 0,1, U	Radical therapy (None 0, Surgery alone 1, RT alone 2, Prostatectomy + postQ RDT 3, Prostatectomy + salvage RDT 4)	mCRPC biopsy site: 1 Bone, 2 node, 3 liver, 4 soft tissue, 5 prostate
V5812	65	1	2	65	4	0	1	0	3
V5652	70	1	1	59	3	1	0	0	2
V5648	62	1	2	250	U	1	1	0	2
V5529	63	1	2	9.4	3	1	0	2	1
V5493	73	1	U	27.42	U	U	U	0	2
V5484	50	1	2	U	3	0	0	3	4
V5330	68	1	2	95	U	U	1	0	4
V5272	59	1	2	129.2	4	1	1	2	4
V5070	58	1	1	2745	1	1	1	0	4
V5288	61	1	1	16.6	1	0	0	2	4

Methods

Immunofluorescence staining

γ-H2AX foci analysis in PCa patient derived explants (PDEs) was done using phosphorylated histone H2A.X (Ser139) antibody (#05-636, Millipore Sigma) using a previously described protocol (1). Explants were treated with JQ1 (1 μM) one day prior to IR (2 Gy) treatment. PDEs were analyzed 1 hr, and 8 hr post IR treatment.

For RAD51 foci analysis, cells were seeded onto Nunc™ Lab-Tek™ II CC2™ 4-well chamber slides (#154917, Thermo Fisher) and probed with primary antibody 4 hr post 6Gy or 10 Gy irradiation following published protocols (2). Chamber slides with cells were first probed with anti-RAD51 primary antibody (#PC130-100ul, Millipore Sigma), then with secondary antibody (Alexa Fluor Plus 594 goat anti rabbit antibody #A32740, Invitrogen/Thermo Fisher). Slides were cured overnight with ProLong™ Gold Antifade Mountant with DAPI (#P36934, Invitrogen/Thermo Fisher). Cells were examined with Deltavision Deconvolution Microscopes (GE Healthcare/Cytiva) using 40x and 60x oil objectives. Foci were counted using Fuji Image J (64 bit for windows); >200 nuclei were scored for each treatment group.

DNA fiber assay

The DNA fiber assays were performed in LNCaP and 22Rv1 cell lines using published protocols (3). Exponentially growing cells were pulse labelled with 100 μM iodo-deoxyuridine (IdU, #I7125-5G, Sigma) for 30 min and 100 μM chloro-deoxyuridine (CldU, #C6891-100mg, Sigma) for 30 min. Subsequently, drug dilutions were administered and incubated for 5 hrs. Mouse anti-BrdU/IdU antibody (#347580, BD Bioscience) and rat anti-BrdU/CldU antibody (#ab6326, Abcam) were used as primary antibodies to probe DNA fibers. Fluorescence-conjugated antibodies including Alexa Fluor 488 anti-rat (#A-11006, Invitrogen/Thermo Scientific) and Alexa Fluor 568 anti-mouse (# A-11004, Invitrogen/Thermo Scientific) were used as secondary antibodies. Fibers were imaged using the Zeiss AxioImager M2 (Carl Zeiss Microscopy) and track length were quantified with Zen 2 (Blue Edition) V2.2 software (Carl Zeiss Microscopy). For statistical analysis, >100 DNA tracks length were quantified for each treatment group.

Cell proliferation assay

Cell proliferation assays were performed using the IncuCyte® S3 Live-Cell Analysis System (Sartorius/ Essen BioScience). Data analysis was conducted using IncuCyte S3 2018B software (Sartorius/ Essen BioScience) together with Graphpad Prism version 8 for windows. Combination Index (CI) was calculated using CompuSyn software (4).

HR & NHEJ flow cytometry assay and HR QPCR assay

HR DNA repair activity was evaluated by transfection of a plasmid harboring an I-SceI endonuclease in engineered HEK293 cells expressing a GFP reporter, DR-GFP, followed by flow cytometry analysis as described elsewhere (5). NHEJ DNA repair activity was evaluated using a flow cytometry protocol as described elsewhere (1).

HR DNA repair activity was also evaluated in LNCaP, VCaP, 22Rv1 and DU145 cells under different treatments using the HR QPCR assay kit (#35600, Nogen Bioteck Corporation) according to the manufacture's protocol as described elsewhere (6). Briefly, cells were first treated with JQ1 (0.1 μM, 1 μM and 10 μM)/DMSO, or transfected with siBRD family/ siNon-targeting for 24 hrs, subsequently followed by co-transfection with the two HR dl plasmids (dl-1 and dl-2). Total DNA was extracted by QIAamp DNA Mini Kit (#51304, Qiagen) 24 hrs post-transfection. QPCR was carried out on QuantStudio 6 Flex Real-Time PCR System (#44856901, ThermoFisher/Applied Biosystems™) to quantify HR DNA repair activity using extracted DNA and the supplied primers according to manufacturer's protocol.

Gene knock-down and gene expression analysis

RNA was extracted from siRNA treated cells using RNeasy Plus Mini Kit (#74136, Qiagen). cDNA synthesis was conducted using the SuperScript VILO cDNA Synthesis Kit (# 11754050, Thermo Fisher/Invitrogen). Primers for BRCA1 (forward primer GAAACCGTGCCAAAAGACTTC, reverse primer CCAAGGTTAGAGAGTTGGACAC) and RAD51 (forward primer CAACCCATTTCACGGTTAGAGC, reverse primer TTCTTTGGCGCATAGGCAACA), BRCA2 (forward primer CACCCACCCTTAGTTCTACTGT, reverse primer CCAATGTGGTCTTTGCAGCTAT), WRN (forward primer CACAGCAGCGGAAATGTCCT, reverse primer GAGCAATCACTAGCATCGTAACT), NBN (forward primer CACCTCCAAAGACAACTGCGGA, reverse primer TCTGTCAGGACGGCAGGAAAGA) and MRE11 (forward primer CAGTGTTTAGTATTCATGGCAATCATG, reverse primer AATGTCCAAGGCACAAAGTGC) are from Primer Bank (7). Source and sequences of primers for GAPDH, BRD2, BRD3 and BRD4 are described elsewhere (1). QRT-PCR was carried out on QuantStudio 6 Flex Real-Time PCR System (#44856901, ThermoFisher/Applied Biosystems™) to verify the knock-down efficiency and/or relative gene expression.

Irradiation of cultured cells

IR was administered to cells using a Shepherd Mark I-68 Cesium-137 irradiator (JL Shepherd). Cells were maintained in a 37°C incubator with 5% CO_2 concentration before and immediately after IR treatment.

Chromatin fractionation assay, Immunoblot analysis and Immunoprecipitation (IP)

Cells were collected 48 hr post transfection of plasmids or 16 hr of BETi treatment and lysed with RIPA buffer supplemented with protease inhibitor cocktail (#11873580001, cOmplete, EDTA free, Roche). Chromatin fractionation was performed as previously described (1). Briefly, cells were harvested after 16 hr of JQ1 (1 uM) treatment and washed with PBS. Cells were suspended for 5 min on ice in the fractionation buffer I (50 mM HEPES, pH 7.5, 150 mM NaCl, 1 mM EDTA, 5 mM Sodium Butyrate) supplemented with 0.2% Nonidet P-40 and protease inhibitors (cOmplete, EDTA free, Roche). Following centrifugation at 1000 g for 5 min, the cytoplasmic supernatant was discarded, then the nuclear pellets were further extracted for 40 min on ice with 150 ul of fractionation buffer II (same as fractionation buffer I, except with 0.5% Nonidet P-40). The pellets containing chromatin were obtained by centrifugation for 15 min at maximum speed at 4°C, and then resuspended in fractionation buffer II.

After determining the concentration of protein, samples were separated on 10% or 12% SDS-PAGE gels and blotted onto PVDF membranes (Millipore Sigma). Membranes were blocked for 1 hr in 5% non-fat dried milk in TBST and incubated for overnight with primary antibody (1:1000) at 4°C. The immunoblots were processed by Supersignal west Pico chemiluminescence kit (#34080, Thermo Scientific), and images were obtained by ChemiDoc Touch Imaging System (Bio-Rad) and processed by Image Lab Touch (Bio-Rad). The following antibodies were used: RAD51 (#PC130, Millipore Sigma), Actin (#AC74, Sigma), BRD4 (#A301-985A, Bethyl), BRD3 (#50818, Abcam), BRD2 (#ab139690, Abcam), TOP1 (#A302-589A, Bethyl), FLAG (#MA1-91878, Thermo Fisher), H2AX (#2595, Cell Signaling).

For immunoprecipitation (IP), cells were lysed with IP Lysis buffer (Thermo Scientific, 25 mM Tris-HCl pH 7.4, 150 mM NaCl, 1 mM EDTA, 1% NP-40 and 5% glycerol) containing protease and phosphatase inhibitor cocktail. The lysates were diluted with an equal volume of the lysis buffer and incubated with 8 µg of BRD4 or TOP1 primary antibody (diluted 1:150) on a rocker for 4 hr at 4 °C. IgG was used as a control. Subsequently, the lysates were incubated with 50 µl of the Dynabeads protein G (#10003D, Thermo Scientific) for another 2 hr at 4 °C. Proteins bound to the beads were washed three times in PBS with 0.01% Tween 20. Elution was performed by heating samples in presence of DTT and protein loading dye. The immunoprecipitated proteins were observed by immunoblot analysis.

RNA extraction, cDNA synthesis and quantitative RT-PCR (QRT-PCR)

Patient tumor RNA were extracted from a 10 µm section of fresh-frozen tumor biopsy samples using AllPrep DNA/RNA/miRNA kit (# 80224, QIAGEN) and from FFPE samples using RNeasy FFPE kit (# 73504, Qiagen). The integrity of the RNA preparations was tested on a TapeStation 4200 (# G2991AA, Agilent), using Agilent High Sensitivity RNA ScreenTape Assay, as suggested by the manufacturer. cDNA synthesis was performed using the SuperScript VILO cDNA Synthesis Kit (# 11754050, Invitrogen) and 200ng of total RNA following manufacturer's instructions. Quantitative-RT-PCR was carried out on 10 matched HSPC/CRPC samples from same patients in quadruplicate, each well containing fluorescent probes for *TOP1*, assay Id Hs01052815_m1 TaqMan Gene Expression Assay (FAM) (Thermofisher) and the control gene *RNA 18S ribosomal 5*; assay Id Hs03928990_g1 TaqMan Gene Expression Assay (VIC). Quantitative reverse transcription PCR (QRT-PCR) was carried out using an AriaDx Real-Time PCR System (Agilent). Fold change in *TOP1* expression levels was calculated by the comparative Ct method, using the formula 2^-(ΔΔCt).

Supplemental References

1. Li X, Baek G, Ramanand SG, Sharp A, Gao Y, Yuan W, et al. BRD4 Promotes DNA Repair and Mediates the Formation of TMPRSS2-ERG Gene Rearrangements in Prostate Cancer. *Cell reports.* 2018;22(3):796-808.
2. Tomimatsu N, Mukherjee B, Catherine Hardebeck M, Ilcheva M, Vanessa Camacho C, Louise Harris J, et al. Phosphorylation of EXO1 by CDKs 1 and 2 regulates DNA end resection and repair pathway choice. *Nat Commun.* 2014;5:3561.
3. Lin YF, Shih HY, Shang ZF, Kuo CT, Guo J, Du C, et al. PIDD mediates the association of DNA-PKcs and ATR at stalled replication forks to facilitate the ATR signaling pathway. *Nucleic acids research.* 2018;46(4):1847-59.
4. Chou TC. Drug combination studies and their synergy quantification using the Chou-Talalay method. *Cancer Res.* 2010;70(2):440-6.
5. Tomimatsu N, Mukherjee B, Harris JL, Boffo FL, Hardebeck MC, Potts PR, et al. DNA-damage-induced degradation of EXO1 exonuclease limits DNA end resection to ensure accurate DNA repair. *J Biol Chem.* 2017;292(26):10779-90.
6. Li L, Karanika S, Yang G, Wang J, Park S, Broom BM, et al. Androgen receptor inhibitor-induced "BRCAness" and PARP inhibition are synthetically lethal for castration-resistant prostate cancer. *Sci Signal.* 2017;10(480).
7. Spandidos A, Wang X, Wang H, and Seed B. PrimerBank: a resource of human and mouse PCR primer pairs for gene expression detection and quantification. *Nucleic acids research.* 2010;38(Database issue):D792-9.
